# Combining hydrophilic and hydrophobic environment sensitive dyes to detect a wide range of cellular polarity†
†Electronic supplementary information (ESI) available: Synthesis, additional methods, and figures (Fig. S1–S32 and Tables S1–S3). See DOI: 10.1039/c9sc04859f


**DOI:** 10.1039/c9sc04859f

**Published:** 2019-11-25

**Authors:** Sang Jun Park, Vinayak Juvekar, Jae Hyung Jo, Hwan Myung Kim

**Affiliations:** a Department of Chemistry , Department of Energy Systems Research , Ajou University , Suwon 443-749 , Korea . Email: kimhm@ajou.ac.kr

## Abstract

Intracellular polarity is an important parameter of pathological and biological phenomena of cells; abnormal polarities are associated with diabetes, neurological diseases, and cancer. However, previously reported polarity probes have issues with quantitatively detecting intracellular polarities, can measure only a limited range of polarities, and can only detect specific intracellular regions. Here, we developed a novel two-dye system, **RPS-1**, that contains a new “turn-on” polarity probe (**Dye1**) based on a spiropyran intramolecular ring closing–opening system activated in polar protic solvents, and a benzothiadiazole containing dye (**Dye3**), which emits only in non-polar solvents with a large stoke shift. Individually, **Dye1** and **Dye3** selectively localized to lysosome and lipid droplets, respectively; however, combining these dyes, which have completely different characteristics, *via* a piperazine linker resulted in the staining of various intracellular organelles. Therefore, as **Dye1** and **Dye3** have the same absorption but different emissions, combining them resulted in a ratiometric polarity probe that could quantitatively measure a wider polarity range inside the cell using a single excitation source. In addition, ratiometric imaging using our **RPS-1** probe to quantitatively detect the distribution of polarity in different cell lines indicated that lysosomes were the most polar organelles in the cell.

## Abstract 2

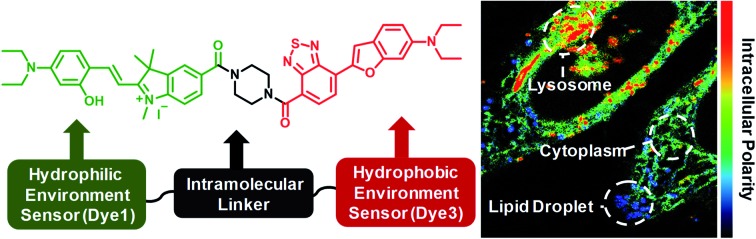
Ratiometric polarity sensitive probe (**RPS-1**) contains two dyes of same absorption but different emissions utilized in comprehensive and quantitative detection of wide range of intracellular polarity.

## Introduction

In the intracellular environment, polarity, viscosity, temperature, redox status, and pH parameters are critical for the initiation and maintenance of the physical and chemical behaviors of biomolecules, because their distribution, spatial arrangement, and composition within the cell are heterogeneous.^1^,^2^ Intracellular polarity, in particular, is key to various cellular processes such as cell proliferation, immune system regulation, increases in the number of local membranes, stimulation of cell migration, and vectorial transport of molecules across the cell layer.^3^,^4^ Each organelle of the cell has an optimal polarity depending on its role and the polarity changes in real time as the intracellular environment changes.^5^–^7^ The pathological activity of the cell changes with its polarity, and abnormal polarity is related to diabetes, neurological diseases, and cancer.^8^–^11^ Therefore, detecting cellular polarity is important in research on pathological and biological phenomena.

The only way to observe intracellular polarity is to use optical imaging; therefore, various fluorescent probes have been developed to detect the polarity of microenvironments.^12^–^27^ Most reported polarity probes are based on intramolecular charge transfer (ICT) and show shifts in emission wavelengths based on changes in the polarity of a cell's surroundings. However, most of these probes are solvatochromic and have disadvantages including the fact that the fluorescence efficiency decreases sharply as the solvent polarity increases, limiting the detection range in hydrophobic environments. In addition, the reported probes can only detect specific structures in a cell, such as the mitochondria,^12^,^13^ lysosomes,^14^–^17^ endoplasmic reticulum (ER),^18^–^20^ lipid droplets,^21^–^23^ and cell membrane.^24^–^26^ Therefore, these probes are limited to detecting the polarity of a particular organelle or a limited zone and have difficulties in imaging the distribution of polarity over the entire cell. Recently, a polarity sensitive probe for detecting lysosomes and lipid droplets was reported.^27^ Even though the probe showed good selectivity, its applications are limited because two different excitation wavelengths must be used to observe each organelle, making it difficult to show the polarity distribution in a cell in real time and thus to quantify the polarity of the organelles. To maintain and regulate appropriate cellular activities, multiple organelles exchange materials and transmit intracellular signals.^28^,^29^ Therefore, studying complex subcellular organelle interactions requires a highly sensitive polarity probe that labels more than two organelles and can quantitatively detect a wide range of cellular polarities.

**Scheme 1:**
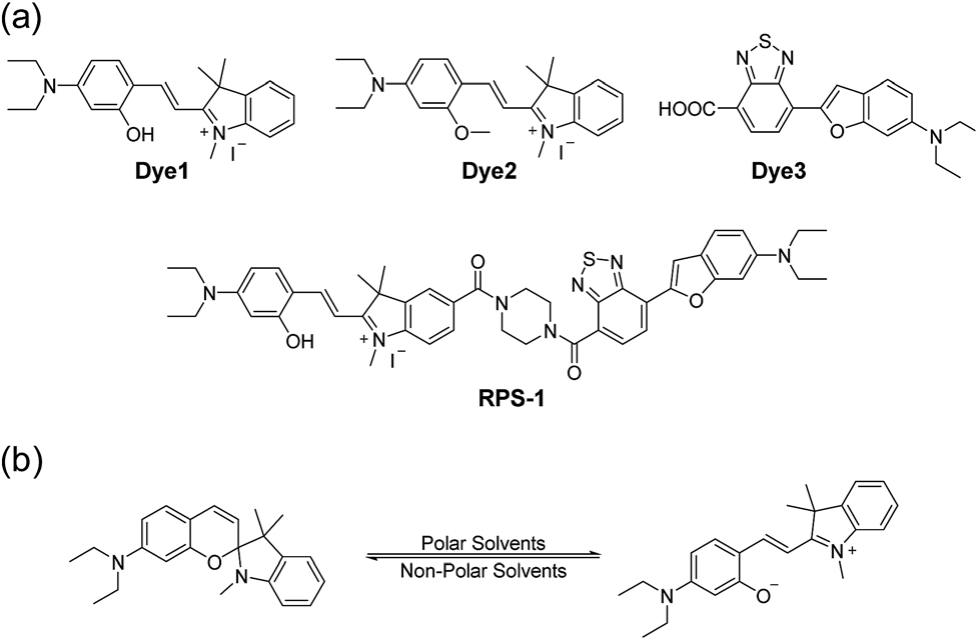
(a) Chemical structures of **Dye1**, **Dye2**, **Dye3**, and **RPS-1**. (b) The proposed polarity sensing mechanism of **Dye1**.

Here, we introduce a ratiometric probe (**RPS-1**) that overcomes the limitations described above. We first synthesized D–π–A type dipolar compounds by treating aminosalicylaldehyde with indolium and a benzothiadiazole acceptor expecting to produce ICT-based dyes, denoted as **Dye1** and **Dye3** (Scheme 1). **Dye3** was characterized as an ICT-based dye whose fluorescence largely shifted to the red-region, but decreased dramatically following an increase in solvent polarity (see below, Fig. S6†). Interestingly, **Dye1** showed an “off–on” characteristic in hydrophobic and hydrophilic solvents as its structure underwent a ring opening/closing cycle. To cover a wide range of polarities, we formed a ratiometric probe that combined these two dyes, which were sensitive to hydrophilic and hydrophobic environments (**Dye1** and **Dye3**), respectively. This probe had different emission windows and could be excited with a single wavelength source. The fluorescence intensity ratio of **RPS-1** was dramatically changed between the yellow and red windows, depending on the polarity, and was highly correlated with the *E*NT value, a parameter that indicates the polarity of a solvent. Therefore, because **RPS-1** could quantitatively detect the polarity of various organelles in cells, the probe revealed that lysosomes were the most polar organelle in cells and that lipid droplets were the most non-polar.

## Results and discussion

Polarity probes were synthesized by condensing salicylaldehyde and 1,2,3,3-tetramethyl-3*H*-indolium iodide under ethanol (EtOH) conditions. The detailed synthesis method, yield, and NMR for each intermediate are described in the ESI.†

**Fig. 1:**
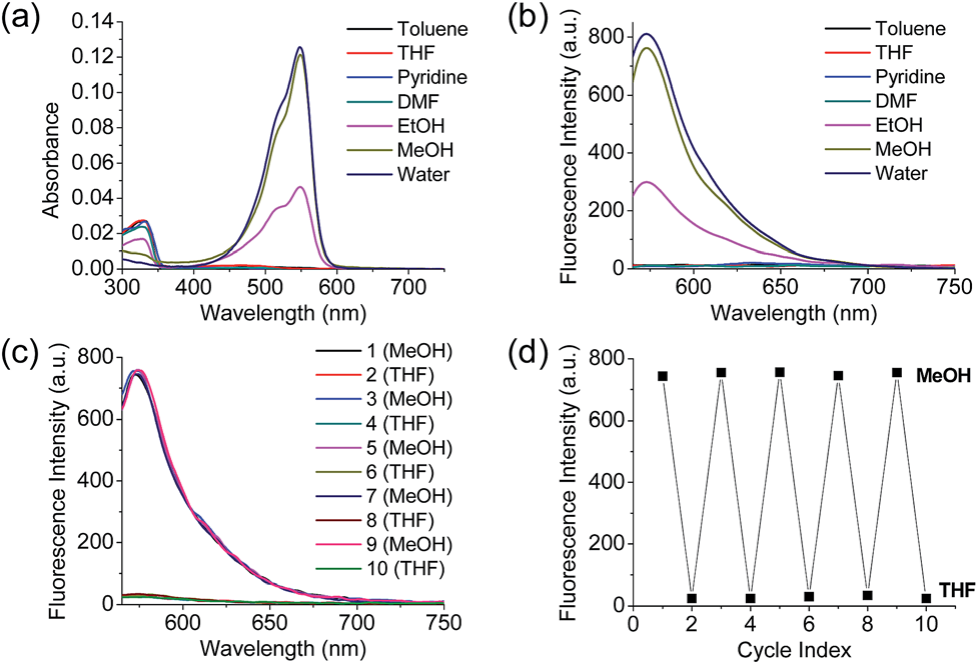
(a) Absorption spectra and (b) fluorescence spectra of **Dye1** in various polar and non-polar solvents. (c) Fluorescence spectrum and (d) fluorescence intensities of **Dye1** with the polarity of MeOH and THF solvents reversed for five cycles. All concentrations for the spectral measurements were 1 μM, the excitation wavelength was 552 nm, and the fluorescence intensity was acquired at 573 nm.

The synthesized **Dye1** was measured for absorbance and fluorescence intensity using solvents with various polarities ranging from toluene to water (Fig. 1). The charge transfer of **Dye1** was blocked in the nonpolar solvent due to its closed-ring structure and it absorbed in the short wavelength region at 328 nm. However, in solvents with a high polarity, such as those above EtOH, **Dye1** opened the ring of the closed structure and increased the conjugation bridge, thus recovering the charge transfer from the diethylaniline to the indolium salt (Scheme 1b). As a result, the absorbance gradually decreased at 328 nm but increased at a long wavelength of 549 nm. Similarly, there was no fluorescence intensity for **Dye1** at an excitation of 552 nm in the nonpolar solvent, but strong fluorescence did occur in the 573 nm region in the highly polar environment.

**Dye2** was synthesized to block cyclization by introducing a methyl group into the hydroxyl group to confirm that the change in absorbance and fluorescence based on the solvent polarity of **Dye1** was the result of the intramolecular cyclization-opening system. **Dye2** showed strong absorbance at approximately 550 nm in all solvents, regardless of their polarity, and showed no absorbance at a wavelength of 328 nm (Fig. S1†). The fluorescence intensities of **Dye2** at the 552 nm excitation source were observed at 580 nm in all the solvents.

To confirm that the intramolecular cyclization and opening of **Dye1** could be reversed by changing the solvent polarity, **Dye1** was added to both MeOH and THF as the solvents five times and the fluorescence spectra were measured (Fig. 1c and d). A constant change in fluorescence was observed under each set of conditions tested; thus, it was confirmed that the intramolecular cyclization and opening for **Dye1** were reversible with the change in solvent polarity.

**Fig. 2:**
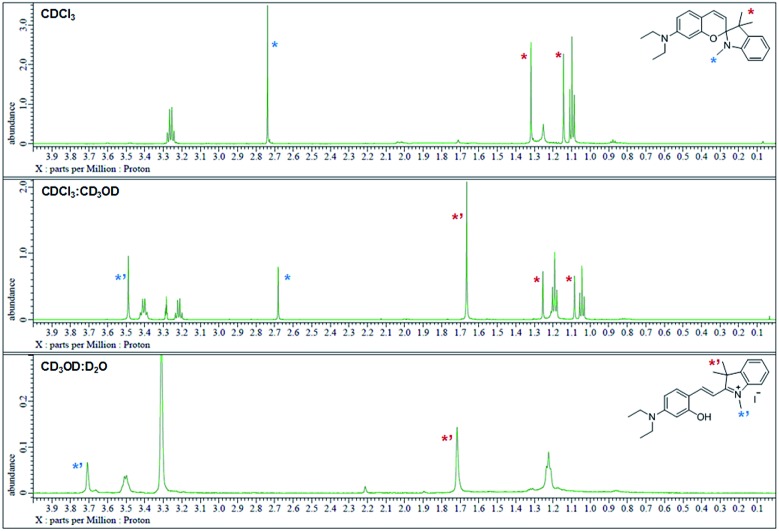
^1^H NMR spectrum (*δ* 0.0–4.0 region) of **Dye1** in CDCl_3_, CDCl_3_ : CD_3_OD = 1 : 1 (v/v) and CD_3_OD : D_2_O = 1 : 1 (v/v) solvents.

The ^1^H NMR spectra were analyzed in nonpolar and polar solvents to further demonstrate the cyclization-opening system of the polarity probes (Fig. 2). In the non-polar CDCl_3_ solvent, **Dye1** was a closed structure and the intramolecular chiral center was present. Due to this, the two *gem*-dimethyl groups in the spirocyclic form, with *δ* 1.14 and 1.32 ppm, were well separated. However, in the polar solvent environment of CD_3_OD : D_2_O = 1 : 1, the spirocyclic form of **Dye1** underwent an intramolecular structural change in the opened structure and the chiral center disappeared. The separated *gem*-dimethyl was observed as one peak at *δ* 1.72 ppm. At the same time, the amine of indoline became positively charged as the polarity of the solvent increased. As a result, the methyl of the amine was shifted from *δ* 2.74 ppm to *δ* 3.71 ppm. In the medium polarity environment of CDCl_3_ : CD_3_OD = 1 : 1, the NMR data showed that both the closed and opened structures coexist. A clear C=C double bond *cis* proton was observed in the aromatic region; it was due to the spirocyclic ring in the nonpolar environment (*J* = 10.3 Hz). In the polar environment, it was difficult to observe a clear *trans* proton but there was a clear and large downfield shift due to the strong electron withdrawing effect. The NMR peaks and chemical structures of **Dye1** for each polarity environment were assigned (Fig. S2†).

The absorbance and fluorescence spectra of the probes in the range of pH 4 to pH 10 were recorded to determine whether the polarity probe was affected by pH (Fig. S3†). The results showed that all polarity probes were always in the open form in aqueous solution, regardless of the change in pH.

**Fig. 3:**
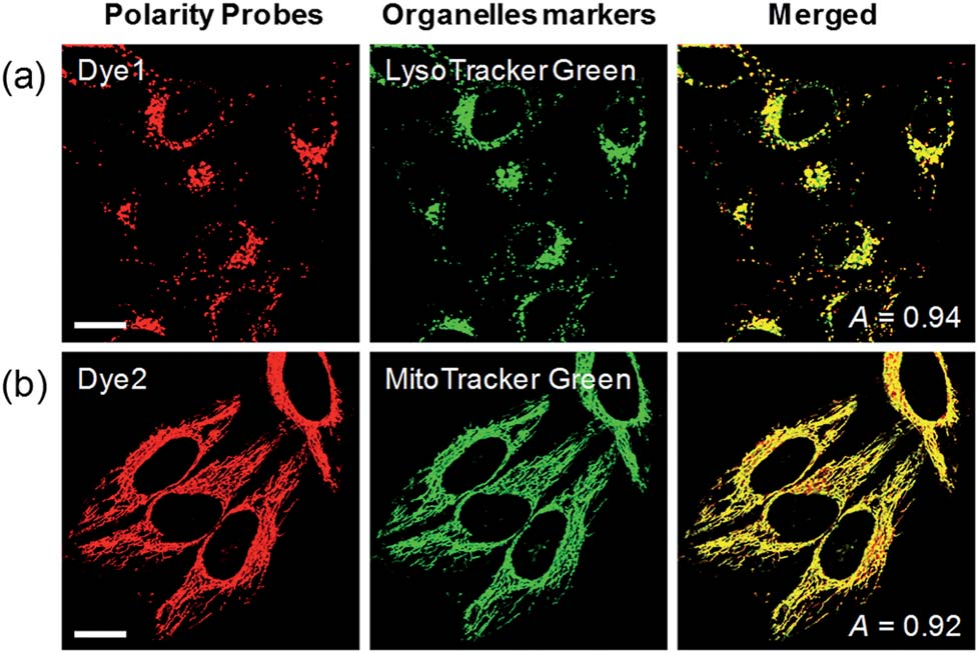
Co-localization assays in HeLa cells for (a) **Dye1** with LysoTracker Green and (b) **Dye2** with Mito-Tracker Green, respectively. Excitation wavelengths were 488 nm (organelle markers) and 552 nm (polarity probes) and the corresponding emissions were recorded at 500–540 nm (organelle markers) and 565–650 nm (polarity probes). Scale bars = 20 μm.

Each polarity probe was incubated in HeLa cells for 30 min. Under the 552 nm excitation wavelength, **Dye1** and **Dye2** emitted bright fluorescence, but the intracellular locations of each probe were different. **Dye1** tended to stain a specific vesicle in the cell, whereas **Dye2**, which was always in the open form, stained a wide area in the cell, which might be mitochondria (Fig. 3). As a result of the co-localization experiments with each of the polarity probes with LysoTracker Green, **Dye1** significantly overlapped with a Pearson coefficient value of 0.94, suggesting that the organelles where this probe was located were lysosomes (Fig. 3a). However, **Dye2** had a low Pearson coefficient with LysoTracker (Fig. S5†), which was not overlapping, and instead showed a high overlap with MitoTracker Green (Fig. 3b). The mitochondrial inner membrane is known to have a proton concentration gradient for ATP synthesis and a negative charge of about –180 mV.^30^ As a result, small molecules with positive charge tend to accumulate inside the mitochondria due to electrostatic attraction. Therefore, **Dye2**, which was positively charged due to the opened structure, was located in the mitochondria for the same reason. However, it has been reported that cyclized indolines like **Dye1** can be located in lysosomes in a tertiary amine form that can act as a proton receptor.^31^ These results suggested that either **Dye1** preferred to be located in the lysosomes or the probe was distributed at various locations within the cell but only the lysosomes could be seen because the lysosomal polarity was specifically higher than other intracellular compartments. The turn-on based probes influenced the fluorescence intensities by various factors such as local concentration, intracellular environment, and imaging conditions. Therefore, to quantitatively confirm the intracellular polarity distribution, a ratiometric probe, whose fluorescence ratio changed according to the environmental polarity, was required.

**Fig. 4:**
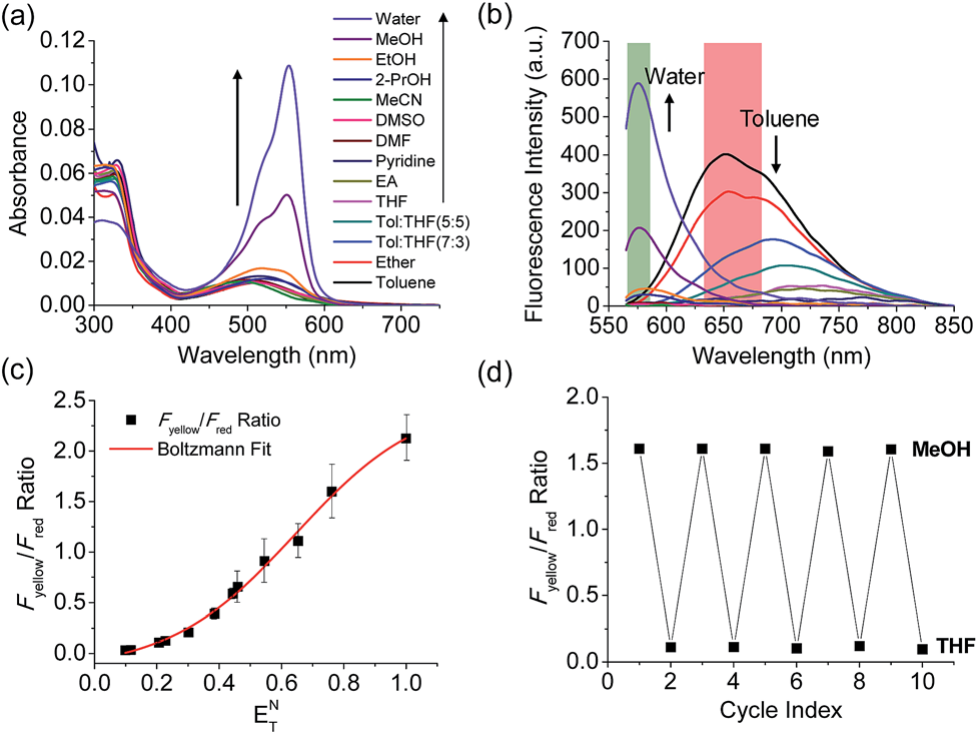
(a) Absorption spectra and (b) fluorescence spectra of **RPS-1** (3 μM) in various polar and non-polar solvents. The green box represents the *F*_yellow_ region and the red box represents the *F*_red_ region. (c) Boltzmann fit of *F*_yellow_/*F*_red_*versus* the orientation polarizability *E*NT. (d) Average *F*_yellow_/*F*_red_ intensity ratios of **RPS-1** with polarity reversibly changed between MeOH and THF solvents for 5 cycles. The excitation wavelength was 552 nm.

The benzothiadiazole derivative (**Dye3**) had an absorption wavelength similar to that of **Dye1**; however, due to a larger stoke shift, the fluorescence emitted in the long wavelength region of the near infrared (NIR) region minimized the fluorescence interference between the two dyes. In contrast to **Dye1**, **Dye3** was a turn-off probe whose fluorescence intensity decreased as the solvent polarity increased (Fig. S6†). **Dye1** and **Dye3** were combined by introducing the piperazine linker, and the two dye fluorescence intensities responded oppositely according to the environmental polarity. Through this, we synthesized a ratiometric polarity probe (**RPS-1**), which had two fluorescence spectra with one excitation source (Scheme 1). **RPS-1** showed a weak absorbance at 510 nm in non-polar solvents such as toluene and ether, based on **Dye3**, and strongly increased the absorbance at 550 nm in polar solvents such as MeOH and water, based on **Dye1** (Fig. 4a). Moreover, as the polarity increased, the fluorescence intensity near 650 nm in the nonpolar solvent gradually decreased and the fluorescence around 580 nm increased (Fig. 4b). The two regions for the ratio fluorescence measurement were defined as 565–585 nm (*F*_yellow_) and 630–680 nm (*F*_red_), and there was a high correlation between the fluorescence ratio of *F*_yellow_/*F*_red_ and the *E*NT value, which is the solvent polarity parameter (*R*^2^ = 0.993, Fig. 4c). The photophysical properties of **RPS-1** are summarized in Table S3.† Additionally, **RPS-1** showed reversible changes in the ratio of *F*_yellow_/*F*_red_ depending on the solvent polarity, a behavior similar to that of **Dye1** (Fig. 4d). The ratio of **RPS-1** was almost constant in various pH ranges (Fig. S7†) and in the presence of other biological metabolites, including reactive oxygen and nitrogen species, cationic and anionic amino acids, glutathione, and enzymes (Fig. S8†). Additionally, **RPS-1** showed high photostability under the imaging conditions used (Fig. S9†), with negligible cytotoxicity (Fig. S4†).

**Fig. 5:**
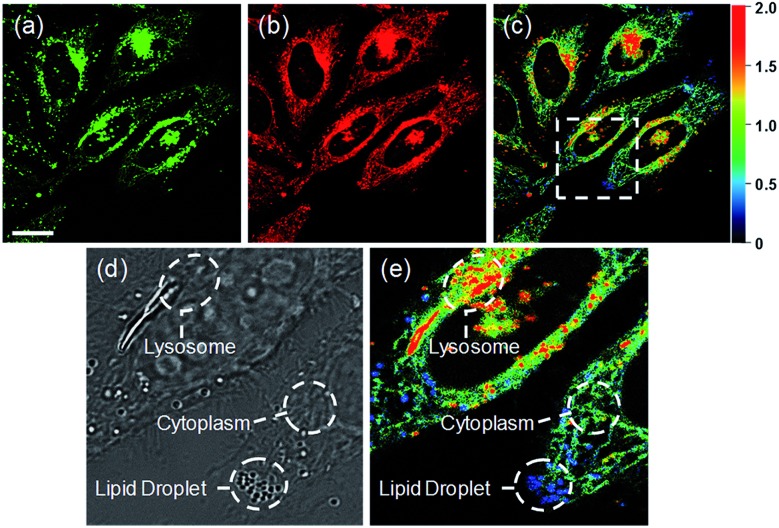
Fluorescence images and pseudocolored ratiometric images (*F*_yellow_/*F*_red_) of HeLa cells labeled with **RPS-1** (3 μM) for 30 min. (a) *F*_yellow_ (565–585 nm) image, (b) *F*_red_ (630–680 nm) image, and (c) ratiometric image. Enlarged (d) bright-field image and (e) ratiometric image of the white box in (c) showing the lysosome, lipid droplet, and cytoplasm. Images were acquired using a 552 nm excitation source. Scale bars = 20 μm.

**Fig. 6:**
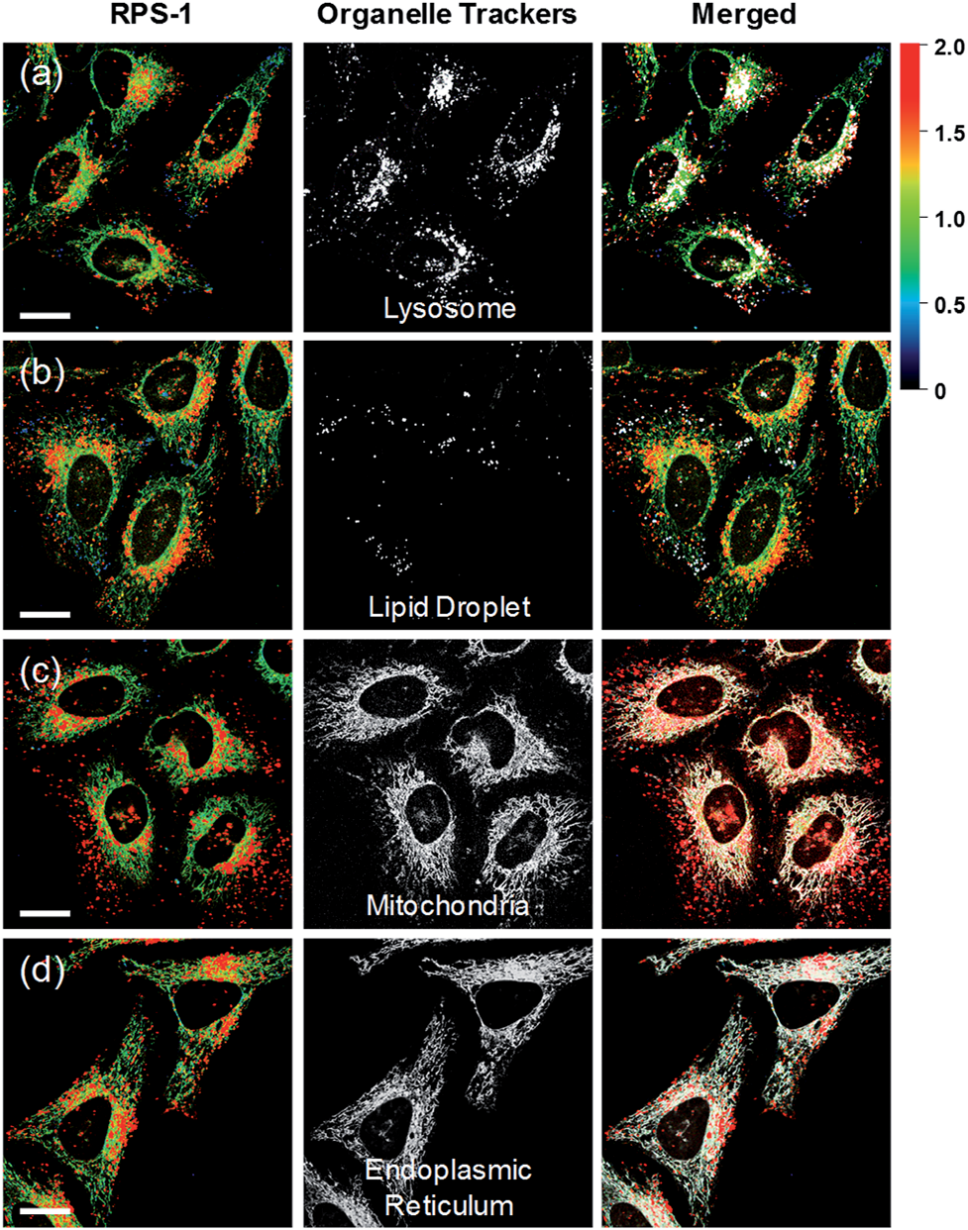
Co-localization assays of **RPS-1** (3 μM) and commercial organelle trackers (1 μM) for (a) lysosomes, (b) lipid droplets, (c) mitochondria, and (d) the endoplasmic reticulum in HeLa cells. Excitation wavelengths were 488 nm (organelle trackers) and 552 nm (**RPS-1**) and the corresponding emissions were recorded at 500–540 nm (organelle trackers), 565–585 nm (**RPS-1**, yellow), and 630–680 nm (**RPS-1**, red). Scale bars = 20 μm.

Ratiometric images were acquired with two channels, *F*_yellow_ and *F*_red_, to confirm whether **RPS-1** reflected the difference in polarity in various compartments within the cell (Fig. 5). The results of HeLa cells labeled with **RPS-1** (3 μM) for 30 min surprisingly showed a clear difference between the fluorescence of the *F*_yellow_ channel and the *F*_red_ channel (Fig. 5a and b). Similar to the cell image of **Dye1**, the *F*_yellow_ channel showed a strong fluorescence in a specific vesicle in the cell. However, the fluorescence of the *F*_red_ channel was observed in other vesicles, in addition to the same *F*_yellow_ vesicle, and stained with strand formation throughout the cell. When the fluorescence images of *F*_yellow_ and *F*_red_ were treated with a pseudocolored ratiometric image (*F*_yellow_/*F*_red_), the distribution of the polarities of each region of the cell could be confirmed at a glance (Fig. 5c). All the intracellular polarity images of **RPS-1** were confirmed by co-localization experiments with various commercial organelle markers (Fig. 6). In the ratiometric images, the red region with the highest polarity overlapped with the lysosome, while the blue region with the lowest polarity overlapped with the lipid droplet marker. The green region with moderate polarity overlapped with both mitochondria and ER markers, and we defined this region as the cytoplasm. In the enlarged images, the lysosome, cytoplasm, and lipid droplet regions were more clearly distinguished, and the blue-colored lipid droplets in the ratiometric image corresponded exactly to the regions of visually observable lipid droplets in the bright-field image (Fig. 5d and e).

**Fig. 7:**
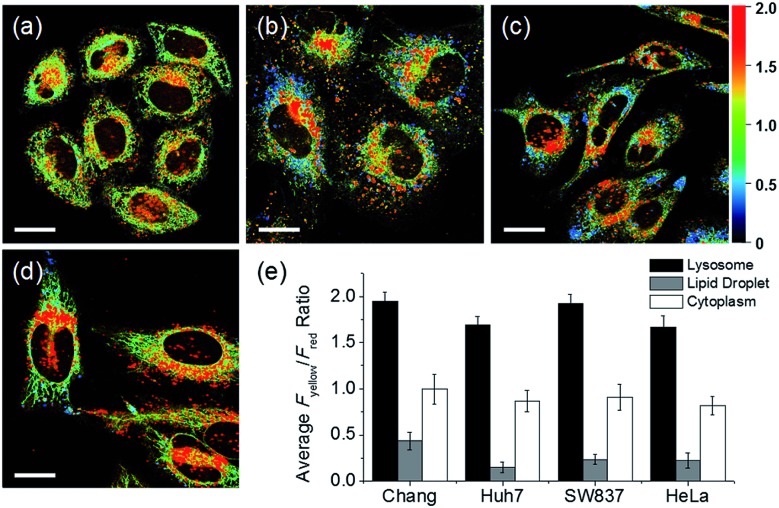
Pseudocolored ratiometric images (*F*_yellow_/*F*_red_) of (a) Chang, (b) Huh7, (c) SW837, and (d) HeLa cells labeled with **RPS-1** (3 μM) for 30 min. (e) Average *F*_yellow_/*F*_red_ intensity ratios of each corresponding image. Images were acquired using 552 nm excitation and emission windows of 565–585 nm (yellow) and 630–680 nm (red). Scale bars = 20 μm.

In addition to HeLa cells, the distribution of intracellular polarity in various cells such as Chang, Huh7, and SW837 was observed; the polarity of the lysosomes was observed to be the highest in all cells (Fig. 7). The *F*_yellow_/*F*_red_ fluorescence ratios of lysosomes in the Chang, Huh7, SW837, and HeLa cells were 1.9, 1.7, 1.9, and 1.7, respectively. The polarity of lysosomes in all cells was as high as the value between those of methanol and water (Fig. 4c). Conversely, the lipid droplets had lower polarity compared to other organelles. The *F*_yellow_/*F*_red_ fluorescence ratio of the lipid droplets in Huh7, SW837, and HeLa cells was 0.15, 0.24, and 0.22, respectively, while that of Chang cells was 0.43, which was higher than that of the other cells. This is consistent with previous studies that showed that the polarity of lipid droplets of cancer cells is lower than that of normal cells due to the specific lipid metabolism of cancer cells.^21^,^32^ The cytoplasmic polarity value was between those of the lysosomes and lipid droplets, and the *F*_yellow_/*F*_red_ fluorescence ratio values in the cytoplasm of Chang, Huh7, SW837, and HeLa were 1.0, 0.87, 0.91, and 0.81, similar to that of 2-PrOH. In particular, the *F*_yellow_/*F*_red_ fluorescence ratios provided information about the polarity of solvents similar to that of lysosomes (1.7–1.9, MeOH and water), cytoplasm (0.81–1.0, 2-PrOH), and lipid droplets (0.15–0.43, DMF and EA). Consequently, intracellular polarity was heterogeneous and the polarity of each organelle gradually decreased from lysosomes, to cytoplasm, to lipid droplets.

## Conclusions

We developed **Dye1**, in which the absorbance and fluorescence intensity changed through intramolecular cyclization-opening according to solvent polarity. **Dye1** is the first hydrophilic environmentally selective turn-on probe in the spiropyran series, in which the structure of the molecule itself changes reversibly in response to polarity. We synthesized a hydrophobic and hydrophilic combined ratiometric probe, **RPS-1**, in which the intensity of the two fluorophores was reversely changed by polarity by introducing **Dye3**, whose fluorescence decreased as the solvent polarity increased. By connecting the two dyes with similar absorption but different solvatochromic shifts, **RPS-1** was able to measure the difference in fluorescence ratios (*F*_yellow_/*F*_red_) according to polarity using one excitation wavelength and quantitatively detect the change in polarity over a wide range, from toluene to water. **RPS-1** was stained in various organelles in the cell and showed a difference in polarities in various regions of the cell at a glance. These results suggested that **RPS-1** could detect a wide range of intracellular polarity changes sensitively and quantitatively, and confirmed that the polarity of the lysosomes was the highest in the cell. This new approach of linking two dyes with completely different characteristics resulted in a new ratiometric polarity sensing dye, **RPS-1**, that could provide useful information to biomedical research.

### Experimental sections

#### Spectroscopic measurements

Absorption spectra and fluorescence spectra were recorded with a UV-Vis spectrophotometer (S-3100) and fluorescence spectrophotometer (FS-2), respectively. The fluorescence quantum yield was measured with 9,10-diphenylanthracene (*Φ* = 0.93 in cyclohexane) as the reference. The ^1^H NMR spectra were recorded using 600 MHz NMR spectrometers (JNM-ECZR). The fluorescence images were obtained with spectral confocal microscopes (Leica TCS SP8).

#### Cell images

Each cell was incubated for two days before imaging. Streptomycin (100 μg mL^–1^), penicillin (100 units per mL), and 10% fetal bovine serum were added to all culture media. The culture medium was replaced with a serum-free medium and each polarity probe was stained for 30 min. Live cell imaging was performed using a live-cell instrument (Chamlide IC) to maintain appropriate temperature, humidity, and pH for long term exposure. Ratiometric image processing and analysis were carried out using MetaMorph software.

#### Co-localization experiments

Experiments were carried out by co-staining with polarity probes and each commercial organelle tracker (LysoTracker Green DND-26 for lysosomes, BODIPY 493/503 for lipid droplets, MitoTracker Green FM for mitochondria and ER-Tracker Green for the endoplasmic reticulum). The excitation wavelengths were 488 nm (organelle trackers) and 552 nm (polarity probes). Pearson's colocalization coefficient (*A*) was calculated using AutoQuant X2 software.

## Conflicts of interest

There are no conflicts to declare.

## Supplementary Material

Supplementary information
