# Supplementary material for: Combining hydrophilic and hydrophobic environment sensitive dyes to detect a wide range of cellular polarity
Source: Chem Sci. 2019 Nov 25;11(2):596–601. doi: 10.1039/c9sc04859f (PMC7069515; doi:10.1039/c9sc04859f)
Supplement: Supplementary file 1 [file SC-011-C9SC04859F-s001.pdf]

Electronic Supplementary Material (ESI) for Chemical Science.
This journal is © The Royal Society of Chemistry 2019

Supporting Information for

Combining hydrophilic and hydrophobic environment sensitive dyes to detect wide range of cellular polarity

Sang Jun Park,‡ Vinayak Juvekar,‡ Jae Hyung Jo and Hwan Myung Kim*

Department of Energy Systems Research, Ajou University, Suwon 443-749, Korea

Fax: +82-31-219-1615, Email: kimhm@ajou.ac.kr

Table of Contents

Page

Synthesis of Dye1, Dye2, Dye3 and RPS-1.. S3
Spectroscopic Measurements.. S7
Cell Viability.. S9
Selectivity Assay.. S12
Photostability.. S13
Figure S1. (a) Absorption spectra and (b) fluorescence spectra of **Dye2** (1 μM) in various polar and non-polar solvents. Excitation wavelength was 552 nm.. S7
Figure S2. ^{1}H NMR spectrum (δ 5.2–9.0 region) of **Dye1** in $CDCl_3$, $CDCl_3$:CD_3OD = 1:1 (v/v) and CD_3OD:D_2O = 1:1 (v/v) solvents.. S8
Figure S3. Effect of pH on the (a,c) absorbance and (b,d) fluorescence spectra for (a,b) **Dye1** and (c,d) **Dye2** in universal buffer (0.1 M citric acid, 0.1 M KH_2PO_4, 0.1 M $Na_2B_4O_7$, 0.1 M Tris, 0.1 M KCl) at 37 °C. Excitation wavelength was 552 nm.. S8
Figure S4. Viability of HeLa cells in the presence of polarity probes as measured by using MTT assays. The cells were incubated with 0–20 μM of probes for 2 h.. S9
Figure S5. Co-localization assays with (a) **Dye2** and (b) LysoTracker Green in HeLa cells. (c) Merged image. Excitation wavelengths were 488 nm (LTG) and 552 nm (**Dye2**) and the corresponding emissions were recorded at 500−540 nm (LTG) and 565−650 nm (**Dye2**), respectively. Scale bars = 20 μm.. S9
Figure S6. (a) Absorption spectra and (b) fluorescence spectra of **Dye3** (3 μM) in various polar and non-polar solvents. Excitation wavelength was 552 nm.. S10
Figure S7. Effect of pH on the (a) absorbance, (b) fluorescence intensity and (c) fluorescence intensity ratios (F_{yellow}/F_{red}) for **RPS-1** in universal buffer (0.1 M citric acid, 0.1 M KH_2PO_4, 0.1 M $Na_2B_4O_7$, 0.1 M Tris, 0.1 M KCl) at 37 °C. Excitation wavelength was 552 nm.. S11
Figure S8. Fluorescence intensity ratio (F_{yellow}/F_{red}) of **RPS-1** (3 μM) with a variety of substances in PBS buffer (10 mM, pH 7.4) at 37 °C [(1) control; 200 μM, (2) TBHP, (3) O^{2-}, (4) $^{\bullet}OH$ (5) $^{\bullet}O^tBu$ (6) H_2O_2, (7) $NO^{\bullet}$, (8) $ONOO^-$; 1 mM, (9) Lys, (10) Arg, (11) His, (12) Asp, (13) Glu, (14) glucose, (15) GSH; 1 unit mL^{-1}, (16) amidase, (17) NTR, (18) ALP, (19)

CE1, (20) CE2, (21) NQO1. The excitation wavelength was 552 nm………………………… S12
Figure S9. Photostability assays in HeLa cells labeled with **RPS-1** (3 μM). (a) Fluorescence images of **RPS-1** before and after irradiation for 60 min. (b) The relative fluorescence intensity as a function of time at 2.00 s intervals for 60 min using *xyt* mode. Images were acquired using 552 nm excitation and emission windows of 565−585 nm (yellow) and 630−680 nm (red). Scale bars = 50 μm………………………………………………………... S13
Figure S10. ^{1}H-NMR spectrum (600 MHz) of **Dye1** in $CDCl_3$…………..………………... S14
Figure S11. ^{13}C-NMR spectrum (150 MHz) of **Dye1** in $CDCl_3$……………………………… S14
Figure S12. HRMS spectrum of **Dye1**………………………………………………………... S15
Figure S13. ^{1}H-NMR spectrum (600 MHz) of **Dye2** in $CDCl_3$……………...……….……... S15
Figure S14. ^{13}C-NMR spectrum (150 MHz) of **Dye2** in $CDCl_3$……………………………… S16
Figure S15. HRMS spectrum of **Dye2**………………..…………………………………… S16
Figure S16. ^{1}H-NMR spectrum (600 MHz) of **4** in $CDCl_3$………..…..…………………… S17
Figure S17. ^{13}C-NMR spectrum (150 MHz) of **4** in $CDCl_3$………………………………... S17
Figure S18. ^{1}H-NMR spectrum (600 MHz) of **6** in $CDCl_3$…………………………………… S18
Figure S19. ^{13}C-NMR spectrum (150 MHz) of **6** in $CDCl_3$………………………………... S18
Figure S20. ^{1}H-NMR spectrum (600 MHz) of **7** in $CDCl_3$…………………………………… S19
Figure S21. ^{13}C-NMR spectrum (150 MHz) of **7** in $CDCl_3$………………………………... S19
Figure S22. ^{1}H-NMR spectrum (600 MHz) of **9** in $CDCl_3$……………………...……… S20
Figure S23. ^{13}C-NMR spectrum (150 MHz) of **9** in $CDCl_3$………………………………... S20
Figure S24. ^{1}H-NMR spectrum (600 MHz) of **10** in $CDCl_3$….…………………….……… S21
Figure S25. ^{13}C-NMR spectrum (150 MHz) of **10** in $CDCl_3$…………..………………... S21
Figure S26. ^{1}H-NMR spectrum (600 MHz) of **11** in $CDCl_3$….…………………….…… S22
Figure S27. ^{13}C-NMR spectrum (150 MHz) of **11** in $CDCl_3$…………..………………... S22
Figure S28. ^{1}H-NMR spectrum (600 MHz) of **Dye3** in DMSO….………………...……… S23
Figure S29. ^{13}C-NMR spectrum (150 MHz) of **Dye3** in DMSO….…………..……………… S23
Figure S30. ^{1}H-NMR spectrum (600 MHz) of **RPS-1** in $CDCl_3$…………………..……. S24
Figure S31. ^{13}C-NMR spectrum (150 MHz) of **RPS-1** in $CDCl_3$…………………………….. S24
Figure S32. HRMS spectrum of **RPS-1**………………...………………………..…… S25
Scheme S1. Synthesis of **Dye1**, **Dye2**, **Dye3** and **RPS-1**……………………………..…. S3
Table S1. Photophysical data for **Dye1**, **Dye2** in polar solvents……………………………… S7
Table S2. Photophysical data for **Dye3** in various solvents…………………………………... S10
Table S3. Photophysical data for **RPS-1** in various solvents……………..…………………..… S11

Synthesis of Dye1, Dye2, Dye3 and RPS-1. Compound **1**, **2**, **3**, **5** and **8** were prepared by the literature methods[1-5] and synthesis of **Dye1**, **Dye2**, **Dye3** and **RPS-1** are described below.

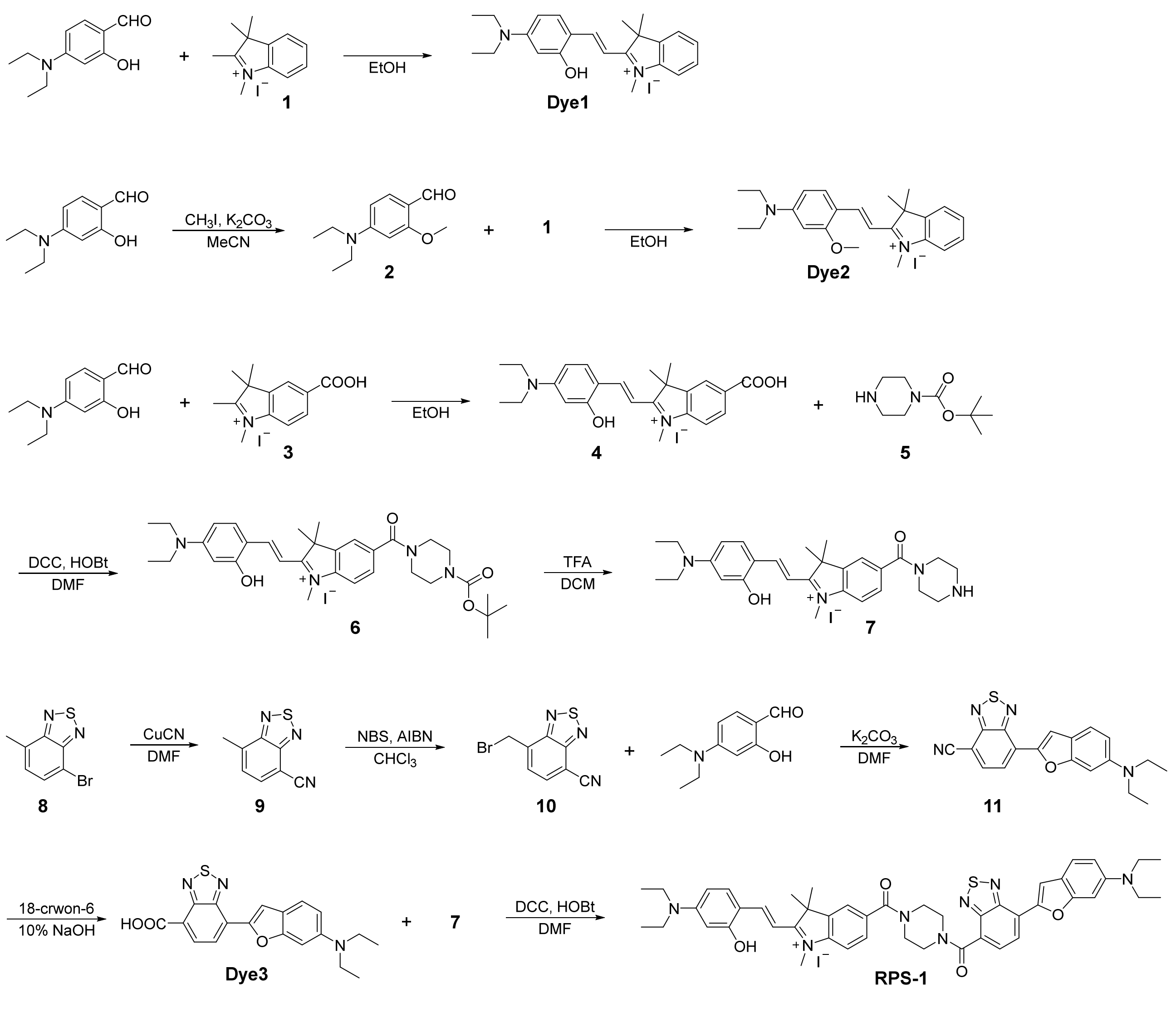

Scheme S1. Synthesis of **Dye1**, **Dye2**, **Dye3** and **RPS-1**.

2-(4-(diethylamino)-2-hydroxystyryl)-1,3,3-trimethyl-3H-indol-1-ium iodide (**Dye1**). A mixture of 4-(diethylamino)-2-hydroxybenzaldehyde (100 mg, 0.517 mmol) and **1** (156 mg, 0.517 mmol) in EtOH (20 mL) was refluxed for 12 h. Then reaction mixture was concentrated under reduced pressure and purified with column chromatography ($CHCl_3$/MeOH = 9:1) to obtain **Dye1** (145 mg) as a dark greenish semi-solid. Yield: 59 %; ^{1}H NMR ($CDCl_3$, 600 MHz): δ (ppm) 7.16 (t, J = 8.3 Hz, 1H), 7.07 (d, J = 6.2 Hz, 1H), 6.85 (d, J = 8.3 Hz, 1H), 6.82 (t, J = 7.9 Hz, 1H), 6.74 (d, J = 10.3 Hz, 1H), 6.52 (d, J = 7.6 Hz, 1H), 6.13 (dd, J = 8.3, 2.8 Hz, 1H), 6.05 (d, J = 2.8 Hz, 1H), 5.37 (d, J = 10.3 Hz, 1H), 3.26 (q, J = 7.1 Hz, 4H), 2.74 (s, 3H), 1.32 (s, 3H), 1.14 (s, 3H), 1.10 (t, J = 7.2 Hz, 6H); ^{13}C NMR ($CDCl_3$, 150 MHz): δ (ppm) 156.17, 149.39, 148.63, 137.35, 129.40, 127.58, 127.47, 121.61, 118.89, 113.53, 107.79, 106.83, 104.61, 103.50, 97.53, 51.45, 44.42, 29.05, 26.07, 20.21, 12.77; HRMS (ESI$^+$): m/z found for $[C_{23}H_{29}ON_2]^+$: 349.2269.

2-(4-(diethylamino)-2-methoxystyryl)-1,3,3-trimethyl-3H-indol-1-ium iodide (**Dye2**). By following synthetic procedure for **Dye1**. **Dye2** was obtained from **1** and **2** as a dark greenish semi-solid. Yield: 61 %; ^{1}H NMR ($CDCl_3$, 600 MHz): δ (ppm) 8.30 (brs, 1H), 8.05 (brs, 1H), 7.35 (d, *J* = 7.6 Hz, 1H), 7.31 (t, *J* = 7.6 Hz, 1H), 7.20-7.26 (m, 2H), 6.98 (d, *J* = 12.4 Hz, 1H), 6.40 (d, *J* = 9.0 Hz, 1H), 5.93 (s, 1H), 3.89 (s, 3H), 3.87 (s, 3H), 3.43 (q, *J* = 6.9 Hz, 4H), 1.60 (s, 6H), 1.14 (t, *J* = 6.9 Hz, 6H); ^{13}C NMR ($CDCl_3$, 150 MHz): δ (ppm) 178.10, 164.17, 155.90, 148.07, 141.85, 141.37, 133.44, 128.92, 126.82, 122.68, 113.16, 112.17, 107.73, 102.41, 92.68, 56.58, 50.25, 45.21, 34.63, 29.48, 28.04, 12.88; HRMS (ESI^{+}): m/z found for $[C_{24}H_{31}ON_2]^+$: 363.2426.

5-carboxy-2-(4-(diethylamino)-2-hydroxystyryl)-1,3,3-trimethyl-3H-indol-1-ium iodide (**4**). By following synthetic procedure for **Dye1**. **4** was obtained from 4-(diethylamino)-2-hydroxybenzaldehyde and **3** as a dark greenish semi-solid. Yield: 58 %; ^{1}H NMR ($CDCl_3$, 600 MHz): δ (ppm) 8.05 (d, *J* = 8.3 Hz, 1H), 7.82 (s, 1H), 6.89 (d, *J* = 9.0 Hz, 1H), 6.79 (d, *J* = 9.6 Hz, 1H), 6.54 (d, *J* = 8.3 Hz, 1H), 6.18 (d, *J* = 9.0 Hz, 1H), 6.06 (s, 1H), 5.37 (d, *J* = 10.3 Hz, 1H), 3.29 (q, *J* = 7.1 Hz, 4H), 2.84 (s, 3H), 1.38 (s, 3H), 1.18 (s, 3H), 1.12 (t, *J* = 7.2 Hz, 6H); ^{13}C NMR ($CDCl_3$, 150 MHz): δ (ppm) 172.13, 155.91, 152.80, 149.74, 137.24, 132.15, 129.83, 127.62, 123.96, 119.26, 112.45, 107.43, 105.78, 104.63, 103.66, 97.34, 50.93, 44.28, 28.71, 25.94, 19.95, 12.74.

5-(4-(tert-butoxycarbonyl)piperazine-1-carbonyl)-2-(4-(diethylamino)-2-hydroxystyryl)-1,3,3-trimethyl-3H-indol-1-ium iodide (**6**). A mixture of **4** (95 mg, 0.182 mmol), N,N'-dicyclohexylcarbodiimide (DCC, 75 mg, 0.363 mmol) and hydroxybenzotriazole (HOBt, 49 mg, 0.363 mmol) in DMF (10 mL) was stirred for 2 h at room temperature. To this mixture, **5** (68 mg, 0.363 mmol) was added and stirred for 12 h. The solvent was evaporated and the reaction mixture was dissolved in CH_3CN then by-product urea was removed by filtration. The filtrate was concentrated under reduced pressure and purified with column chromatography ($CHCl_3$/MeOH = 9:1) to obtain **6** (81 mg) as a dark greenish semi-solid. Yield: 65 %; ^{1}H NMR ($CDCl_3$, 600 MHz): δ (ppm) 7.24 (dd, *J* = 8.3, 1.4 Hz, 1H), 7.14 (d, *J* = 1.4 Hz 1H), 6.83 (d, *J* = 8.3 Hz, 1H), 6.72 (d, *J* = 9.6 Hz, 1H), 6.44 (d, *J* = 8.3 Hz, 1H), 6.12 (dd, *J* = 8.3, 2.1 Hz, 1H), 5.98 (d, *J* = 2.1 Hz 1H), 5.31 (d, *J* = 9.6 Hz, 1H), 3.61 (brs, 4H), 3.44 (brs, 4H), 3.24 (q, *J* = 7.3 Hz, 4H), 2.74 (s, 3H), 1.45 (s, 9H), 1.29 (s, 3H), 1.11 (s, 3H), 1.08 (t, *J* = 6.9 Hz, 6H); ^{13}C NMR ($CDCl_3$, 150 MHz): δ (ppm) 171.74, 155.86, 154.74, 150.35, 149.49, 137.49, 129.68, 128.15, 127.58, 125.08, 121.76, 112.88, 107.59, 105.71, 104.61, 103.81, 97.41, 80.24, 51.32, 44.39, 34.01, 28.88, 28.47, 26.00, 20.16, 12.75.

2-(4-(diethylamino)-2-hydroxystyryl)-1,3,3-trimethyl-5-(piperazine-1-carbonyl)-3H-indol-1-ium iodide (**7**). A compound **6** (90 mg, 0.131 mmol) was dissolved in a co-solvent of TFA (2 mL) and

DCM (2 mL) then stirred for 12 h at room temperature. Then reaction mixture was washed with 10 % Na_2CO_3 (10 mL) and brine (10 mL × 2). The organic phase was concentrated under reduced pressure and purified with column chromatography ($CHCl_3$/MeOH = 8:2) to obtain **7** (69 mg) as a dark greenish semi-solid. Yield: 90 %; ^1H NMR ($CDCl_3$, 600 MHz): δ (ppm) 7.26 (d, J = 9.0 Hz, 1H), 7.16 (s, 1H), 6.86 (d, J = 8.3 Hz, 1H), 6.75 (d, J = 10.3 Hz, 1H), 6.46 (d, J = 7.6 Hz, 1H), 6.14 (d, J = 9.0 Hz, 1H), 6.00 (s, 1H), 5.32 (d, J = 9.6 Hz, 1H), 3.85 (d, J = 5.5 Hz, 4H), 3.27 (q, J = 7.3 Hz, 4H), 3.08 (s, 4H), 2.77 (s, 3H), 1.30 (s, 3H), 1.13 (s, 3H) 1.10 (t, J = 7.2 Hz, 6H); ^{13}C NMR ($CDCl_3$, 150 MHz): δ (ppm) 171.60, 155.88, 150.27, 149.45, 137.47, 129.68, 128.10, 127.58, 125.13, 121.73, 112.91, 107.57, 105.74, 104.62, 103.75, 97.39, 51.35, 45.83, 44.41, 31.02, 28.92, 26.02, 20.18, 12.75.

7-methylbenzo[c][1,2,5]thiadiazole-4-carbonitrile (**9**). To a solution of **8** (1.00 g, 4.38 mmol) in DMF (15 mL) in cylindrical glass pressure tube CuCN (1.23 g, 13.7 mmol) was added and stirring at 150 °C 16 h. After cooling to room temperature aqueous 15 % NH_3 (50 mL) was added and stirred for 1 h. The resulting precipitate was filtrated and washed with DCM. The filtrate was extracted three times with DCM, organic solvent was evaporated, and the product was purified on a silica gel column by using DCM as eluent to obtain **9** (0.57 g) as a white fluffy solid. Yield: 74 %; ^1H NMR ($CDCl_3$, 600 MHz): δ (ppm) 7.94 (d, J = 6.9 Hz, 1H), 7.44 (d, J = 8.3 Hz, 1H), 2.82 (s, 3H); ^{13}C NMR ($CDCl_3$, 150 MHz): δ (ppm) 154.85, 152.90, 138.58, 136.20, 127.46, 115.67, 103.44, 18.56.

7-(bromomethyl)benzo[c][1,2,5]thiadiazole-4-carbonitrile (**10**). To a stirred solution of **9** (1.36 g, 7.78 mmol), 2,2'-azobis(2-methylpropionitrile) (AIBN, 50 mg, 0.304 mmol) and N-bromosuccinimide (NBS, 1.66 g, 9.33 mmol) in dry $CHCl_3$ (60 mL) added 0.5 mL of 33 % HBr in acetic acid and stirred at 75 °C for 16 h. After reaction completion monitored by TLC, the mixture was dissolved in $CHCl_3$ (100 mL) and washed with water, organic layer dried over Na_2SO_4, filter and concentrated. The crude product was purified by column chromatography (hexane/EA = 9:1) to obtain **10** (1.53 g) as a white solid. Yield: 78 %; ^1H NMR ($CDCl_3$, 600 MHz): δ (ppm) 8.02 (d, J = 7.6 Hz, 1H), 7.74 (d, J = 7.6 Hz, 1H), 4.98 (s, 2H); ^{13}C NMR ($CDCl_3$, 150 MHz): δ (ppm) 153.11, 152.87, 136.70, 135.94, 128.46, 115.09, 106.02, 26.84.

7-(6-(diethylamino)benzofuran-2-yl)benzo[c][1,2,5]thiadiazole-4-carbonitrile (**11**). A mixture of **10** (500 mg, 1.97 mmol), 4-(diethylamino)salicylaldehyde (390 g, 2.02 mmol), and K_2CO_3 (1.70 g, 12.3 mmol) was stirred in DMF (10 mL) for 16 h at 125 °C. The dark reaction mixture was diluted with water and extracted with EA. The organic solvent was evaporated, and the product was purified on column chromatography (hexane/EA = 9:1) to obtain **11** (151 mg) as a dark violet solid.

Yield: 23 %; ^{1}H NMR ($CDCl_3$, 600 MHz): δ (ppm) 8.17 (s, 1H), 8.05 (d, *J* = 7.6 Hz, 1H), 8.02 (d, *J* = 7.6 Hz, 1H), 7.48 (d, *J* = 8.3 Hz, 1H), 6.75 (s, 1H), 6.73 (dd, *J* = 8.3, 2.1 Hz, 1H), 3.45 (q, *J* = 7.1 Hz, 4H), 1.24 (t, *J* = 7.2 Hz, 6H); ^{13}C NMR ($CDCl_3$, 150 MHz): δ (ppm) 158.28, 153.88, 150.33, 148.37, 147.80, 136.36, 128.30, 122.81, 121.10, 118.71, 116.17, 113.62, 110.44, 101.74, 92.81, 45.10, 12.71.

7-(6-(diethylamino)benzofuran-2-yl)benzo[c][1,2,5]thiadiazole-4-carboxylic acid (**Dye3**). A compound **11** (100 mg, 0.287 mmol) and 18-crown-6 (100 mg, 0.379 mmol) was dissolved in a co-solvent of 10 % NaOH (5 mL) and 1,4-dioxane (5 mL) then stirred for overnight at 100 °C. The solvent was evaporated and the reaction mixture is acidified to pH = 2 with HCl in an ice bath. After diluting with EA (20 mL), the organic phase was concentrated under reduced pressure and purified with column chromatography (DCM/methanol = 94:6) to obtain **9** (31 mg) as a dark purple semi-solid. Yield: 29 %; ^{1}H NMR (DMSO, 600 MHz): δ (ppm) 8.35 (d, *J* = 6.2 Hz, 1H), 8.07 (s, 1H), 8.06 (d, *J* = 6.6 Hz, 1H), 7.51 (d, *J* = 8.2 Hz, 1H), 6.81 (s, 1H), 6.73 (d, *J* = 9.0 Hz, 1H), 3.39 (q, *J* = 7.3 Hz, 4H), 1.11 (t, *J* = 6.9 Hz, 6H); ^{13}C NMR ($CDCl_3$, 150 MHz): δ (ppm) 165.86, 157.80, 152.95, 151.31, 148.17, 134.40, 126.83, 123.16, 122.01, 121.59, 118.23, 112.10, 110.65, 92.99, 44.78, 12.97.

2-(4-(diethylamino)-2-hydroxystyryl)-5-(4-(7-(6-(diethylamino)benzofuran-2-yl)benzo[c][1,2,5]thiadiazole-4-carbonyl)piperazine-1-carbonyl)-1,3,3-trimethyl-3H-indol-1-ium iodide (**RPS-1**). A mixture of **Dye3** (12 mg, 0.028 mmol), DCC (11 mg, 0.55 mmol) and HOBt (7 mg, 0.055 mmol) in DMF (3 mL) was stirred for 2 h at room temperature. To this mixture, **7** (15 mg, 0.028 mmol) was added and stirred for 12 h. The solvent was evaporated and the reaction mixture was dissolved in CH_3CN then by-product urea was removed by filtration. The filtrate was concentrated under reduced pressure and purified with column chromatography ($CHCl_3$/MeOH = 9:1) to obtain **RPS-1** (9 mg) as a dark purple semi-solid. Yield: 34 %; ^{1}H NMR ($CDCl_3$, 600 MHz): δ (ppm) 8.11 (d, *J* = 7.6 Hz, 1H), 8.05 (s, 1H), 7.76 (d, *J* = 6.9 Hz, 1H), 7.48 (d, *J* = 9.0 Hz, 1H), 7.29 (d, *J* = 8.3 Hz, 1H), 7.48 (d, *J* = 9.0 Hz, 1H), 6.85 (d, *J* = 9.0 Hz, 1H), 6.80 (s, 1H), 6.74 (d, *J* = 10.8 Hz, 1H), 6.72 (d, *J* = 8.4, 1H) 6.47 (d, *J* = 8.3 Hz, 1H), 6.13 (s, 1H), 6.00 (d, *J* = 9.0 Hz, 1H), 5.32 (d, *J* = 10.3 Hz, 1H), 3.64–3.96 (m, 8H), 3.44 (q, *J* = 7.1 Hz, 4H), 3.25 (q, *J* = 7.3 Hz, 4H), 2.76 (s, 3H), 1.30 (s, 3H), 1.23 (t, *J* = 7.6 Hz, 6H), 1.13 (s, 3H), 1.09 (t, *J* = 7.5 Hz, 6H); 13C NMR ($CDCl_3$, 150 MHz): δ (ppm) 171.85, 166.77, 157.66, 155.85, 152.15, 150.87, 150.52, 149.48, 148.52, 147.72, 137.56, 129.71, 129.54, 128.32, 127.58, 126.27, 125.38, 124.69, 122.31, 121.85, 118.82, 112.82, 110.90, 110.11, 107.54, 105.76, 104.61, 103.77, 97.39, 93.33, 51.33, 45.10, 44.41, 29.78, 28.91, 26.02, 22.78, 20.17, 14.22, 12.74 HRMS (ESI$^+$): m/z found for $[C_{47}H_{52}O_4N_7S]^+$: 810.3788.

Spectroscopic Measurements. Absorption spectra and fluorescence spectra were recorded with UV-Vis spectrophotometer (S-3100) and fluorescence spectrophotometer (FS-2), respectively. The fluorescence quantum yield was measured with 9,10-diphenylanthrancene (Φ = 0.93 in cyclohexane) as the reference. ^{1}H NMR spectra was recorded using 600 MHz NMR spectrometers (JNM-ECZR). Fluorescence images were obtained with spectral confocal microscopes (Leica TCS SP8).

Table S1. Photophysical data for **Dye1** and **Dye2** in polar solvents.

Compound	Solvent	λ_{max}^{abs} (nm)^a	λ_{max}^{fl} (nm)^b	Φ (%)^c	ε ($M^{-1}cm^{-1}$)^d
**Dye1**	EtOH	549	573	0.55	4.64 × 10^4
	MeOH	549	573	0.47	12.12 × 10^4
	Water	549	573	0.46	12.55 × 10^4
**Dye2**	EtOH	556	580	1.4	8.57 × 10^4
	MeOH	553	579	0.73	8.30 × 10^4
	Water	549	579	1.1	6.34 × 10^4

a) Maximum absorption wavelength in absorption spectra. b) Maximum emission wavelength in fluorescence spectra. c) Fluorescence quantum yield. d) Molar extinction coefficients.

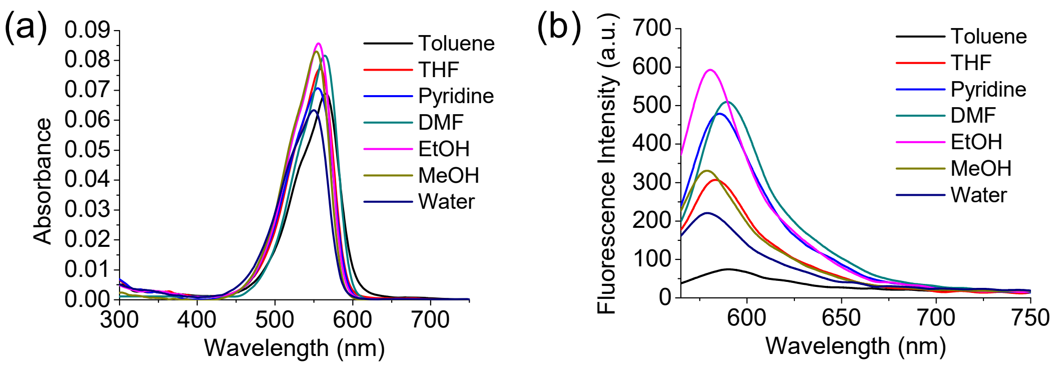

Figure S1. (a) Absorption spectra and (b) fluorescence spectra of **Dye2** (1 μM) in various polar and non-polar solvents. Excitation wavelength was 552 nm.

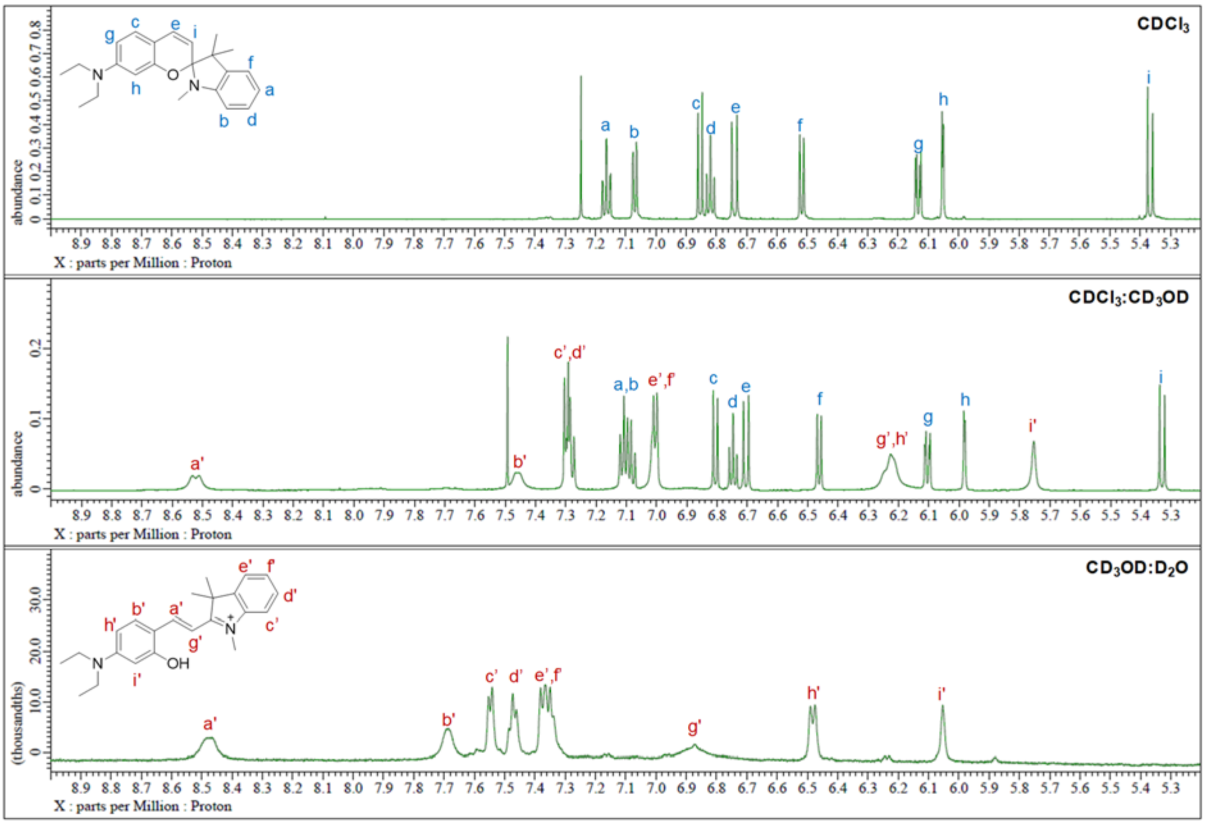

Figure S2. ^{1}H NMR spectrum (δ 5.2–9.0 region) of **Dye1** in $CDCl_3$, $CDCl_3$:CD_3OD = 1:1 (v/v) and CD_3OD:D_2O = 1:1 (v/v) solvents.

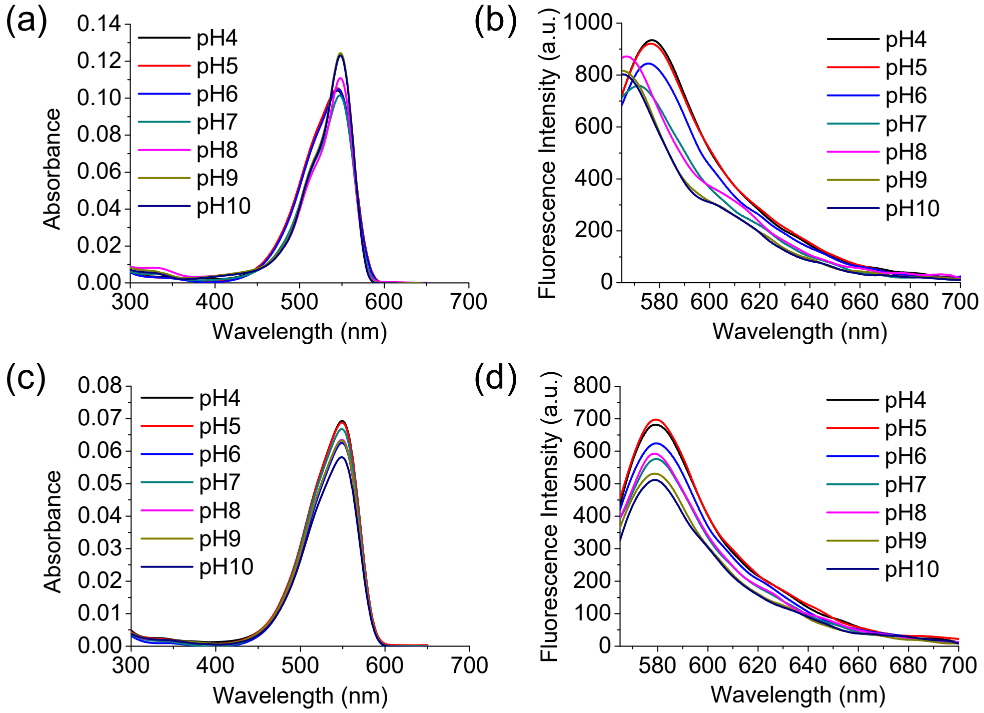

Figure S3. Effect of pH on the (a,c) absorbance and (b,d) fluorescence spectra for (a,b) **Dye1** and (c,d) **Dye2** in universal buffer (0.1 M citric acid, 0.1 M KH_2PO_4, 0.1 M $Na_2B_4O_7$, 0.1 M Tris, 0.1 M KCl) at 37 °C. Excitation wavelength was 552 nm.

Cell Viability. MTT kit (AbCareBio CL) assay was performed to assess the cytotoxicity. HeLa cells were cultured in 96-well plate for 24 h, and then each different concentration of probes was added. After incubation for 2 h, the cultured medium was replaced with serum free medium containing 10 % MTT, and further incubated for 2 h. MTT containing medium was removed and DMSO was added to dissolve the formed formazan precipitate. Absorbance was measured at 600 nm.

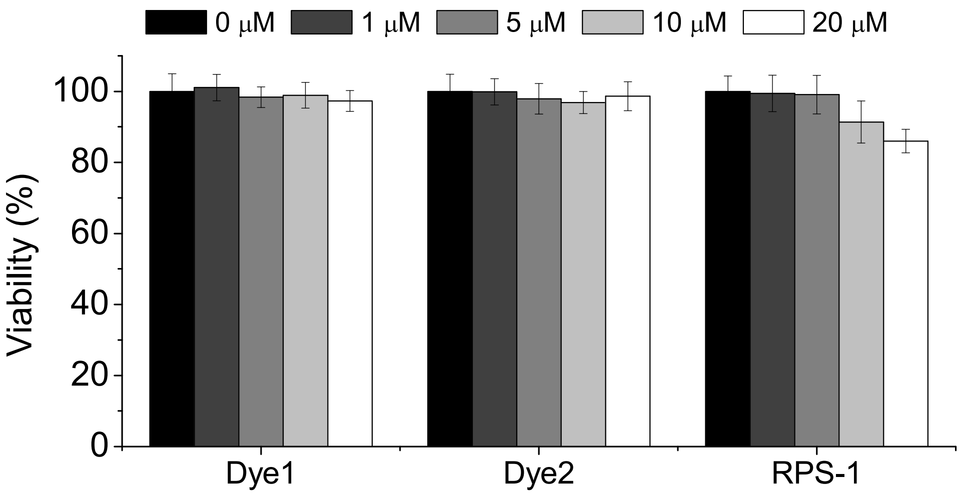

Figure S4. Viability of HeLa cells in the presence of polarity probes as measured by using MTT assays. The cells were incubated with 0–20 μM of probes for 2 h.

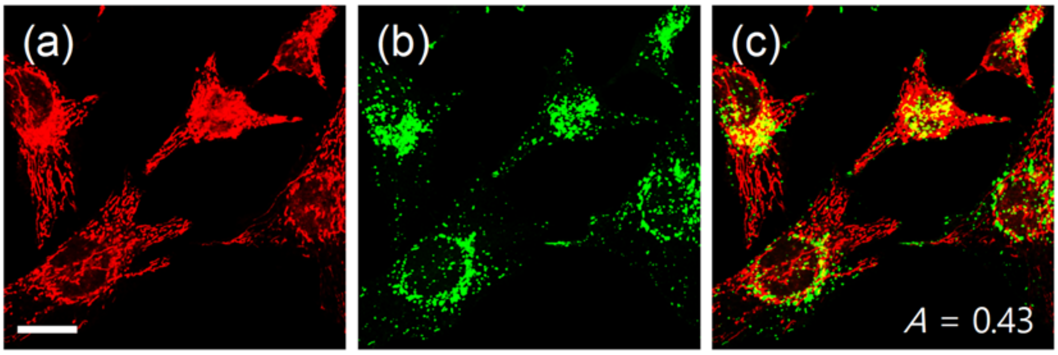

Figure S5. Co-localization assays with (a) **Dye2** and (b) LysoTracker Green in HeLa cells. (c) Merged image. Excitation wavelengths were 488 nm (LTG) and 552 nm (**Dye2**) and the corresponding emissions were recorded at 500−540 nm (LTG) and 565−650 nm (**Dye2**), respectively. Scale bars = 20 μm.

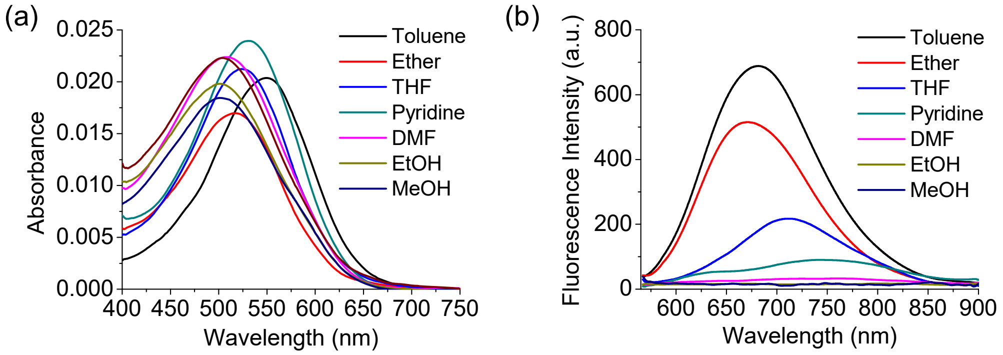

Figure S6. (a) Absorption spectra and (b) fluorescence spectra of **Dye3** (3 μM) in various polar and non-polar solvents. Excitation wavelength was 552 nm.

Table S2. Photophysical data for **Dye3** in various solvents.

Compound	Solvent	λ_{max}^{abs} (nm)[a]	λ_{max}^{fl} (nm)[b]	Φ (%)[c]	ε ($M^{-1}cm^{-1}$)[d]
**Dye3**	Toluene	549	681	6.4	6.79×10^3
	Ether	515	671	6.0	5.66×10^3
	THF	524	712	2.2	7.08×10^3
	Pyridine	533	742	0.81	7.98×10^3
	DMF	510	759	0.36	7.46×10^3
	EtOH	503	ND	ND	6.15×10^3
	MeOH	503	ND	ND	7.43×10^3

a) Maximum absorption wavelength in absorption spectra. b) Maximum emission wavelength in fluorescence spectra. c) Fluorescence quantum yield. d) Molar extinction coefficients.

Table S3. Photophysical data for **RPS-1** in various solvents.

Solvent	λ_{max}^{abs} (nm)[a]	λ_{max}^{fl} (nm)[b]	Φ (%)[c]	ε ($M^{-1}cm^{-1}$)[d]	E_T^{Ne}	Ratio[f]
Toluene	512	651	17.	3.51×10^3	0.099	0.0291
Ether	512	654	11.	3.49×10^3	0.117	0.0331
THF	507	735	1.5	3.59×10^3	0.207	0.106
EA	498	720	2.0	3.25×10^3	0.228	0.123
Pyridine	516	763	1.1	4.45×10^3	0.302	0.207
DMF	501	586	0.49	3.94×10^3	0.386	0.390
DMSO	505	588	0.76	3.57×10^3	0.444	0.586
MeCN	491	590	0.27	3.66×10^3	0.46	0.653
2-PrOH	520	583	1.1	4.01×10^3	0.546	0.909
EtOH	520	580	0.71	5.62×10^3	0.546	1.108
MeOH	551	577	0.24	1.68×10^4	0.762	1.597
Water	552	576	0.28	3.63×10^4	1	2.125

a) Maximum absorption wavelength in absorption spectra. b) Maximum emission wavelength in fluorescence spectra. c) Fluorescence quantum yield. d) Molar extinction coefficients. e) Normalized solvent polarity scale. f) Fluorescence emission ratio (F_{yellow}/F_{red}) in fluorescence spectra.

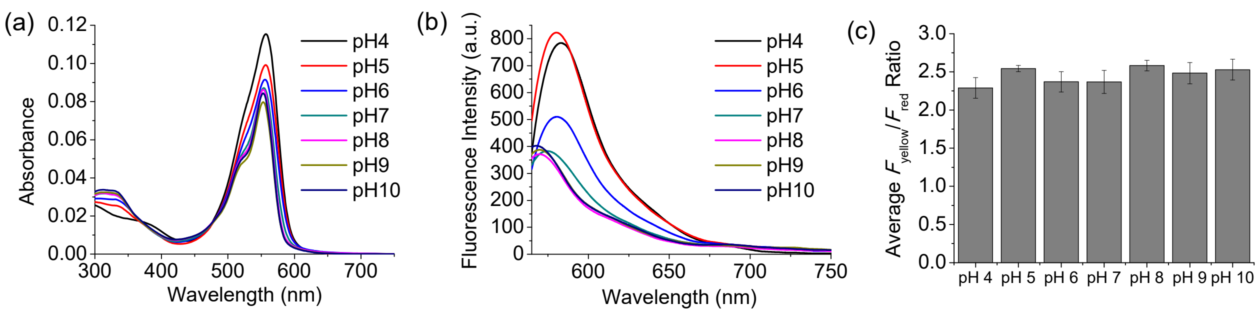

Figure S7. Effect of pH on the (a) absorbance, (b) fluorescence spectra and (c) fluorescence intensity ratios (F_{yellow}/F_{red}) for **RPS-1** in universal buffer (0.1 M citric acid, 0.1 M KH_2PO_4, 0.1 M $Na_2B_4O_7$, 0.1 M Tris, 0.1 M KCl) at 37 °C. Excitation wavelength was 552 nm.

Selectivity Assay. Each species (200 μM of ROS and RNS; 1mM of amino acids, glucose and GSH; 1 unit mL^{-1} of enzymes) were administered to 3 μM of **RPS-1** in PBS buffer (10 mM, pH 7.4) and the fluorescence spectra were measured as time. Temperature was maintained for 2 h at 37 °C. *tert*-butyl hydroperoxide (TBHP, 416665), KO_2 (278904), amino acids (LAA21), glucose (G7528), glutathione (GSH, G6013), amidase (A6691), nitroreductase (NTR, N9284), alkaline phosphatase (ALP, P7640), carboxylesterase 1 (CE1, E0287), carboxylesterase 2 (CE2, E0412), quinone reductase (NQO1, D1315) were purchased from Sigma- Aldrich. Hydroxyl radical (•OH) and *tert*-butoxyl radical (•O^{t}Bu) were generated from TBHP and H_2O_2 by $FeSO_4$. Peroxynitrite ($ONOO^-$) was prepared following the reported method.[6]

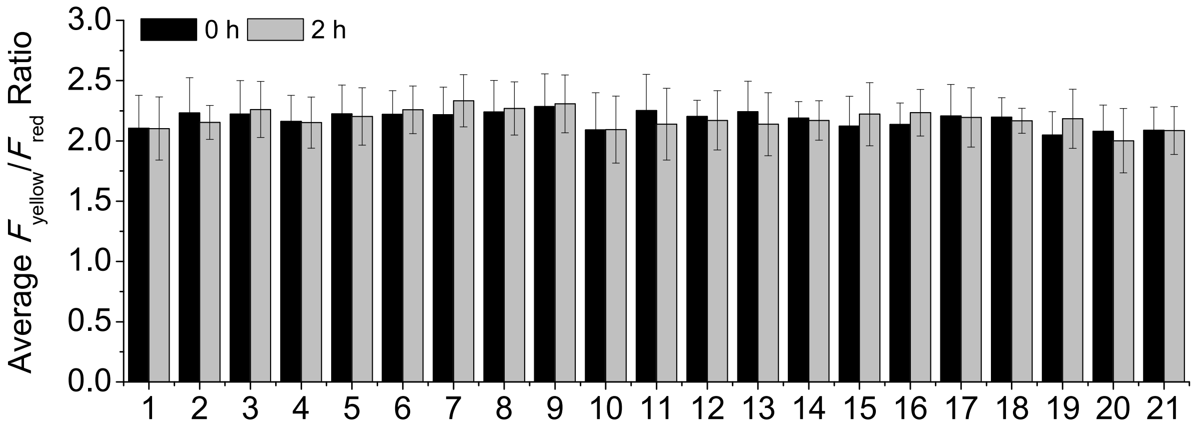

Figure S8. Fluorescence intensity ratio (F_{yellow}/F_{red}) of **RPS-1** (3 μM) with a variety of substances in PBS buffer (10 mM, pH 7.4) at 37 °C [(1) control; 200 μM, (2) TBHP, (3) O_2^-, (4) •OH (5) •O^{t}Bu (6) H_2O_2, (7) NO•, (8) $ONOO^-$; 1 mM, (9) Lys, (10) Arg, (11) His, (12) Asp, (13) Glu, (14) glucose, (15) GSH; 1 unit mL^{-1}, (16) amidase, (17) NTR, (18) ALP, (19) CE1, (20) CE2, (21) NQO1. The excitation wavelength was 552 nm.

Photostability. Photostability of **RPS-1** was measured as the variation in fluorescence intensity over time at three designated positions in **RPS-1**-labeled (3 μM) HeLa cells. Fluorescence intensity was unchanged for 60 min, indicating high photostability.

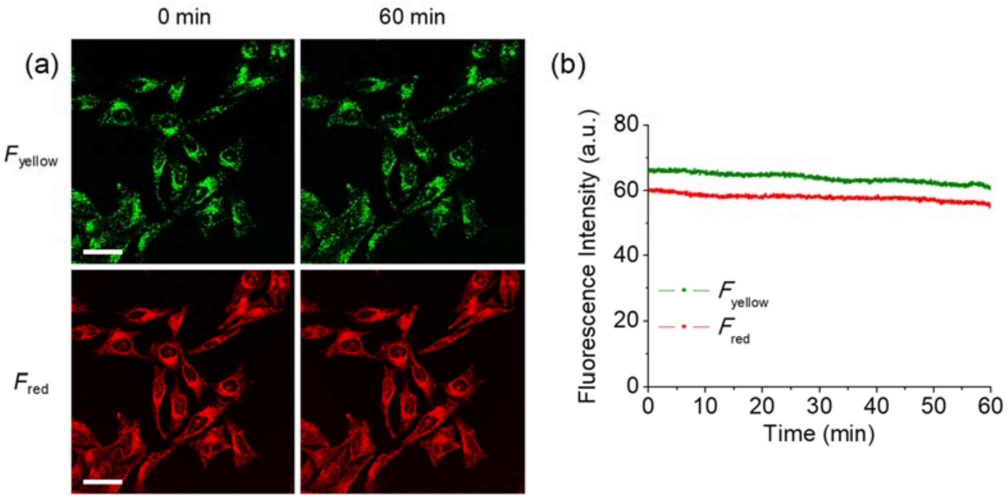

Figure S9. Photostability assays in HeLa cells labeled with **RPS-1** (3 μM). (a) Fluorescence images of **RPS-1** before and after irradiation for 60 min. (b) The relative fluorescence intensity as a function of time at 2.00 s intervals for 60 min using *xyt* mode. Images were acquired using 552 nm excitation and emission windows of 565−585 nm (yellow) and 630−680 nm (red). Scale bars = 50 μm.

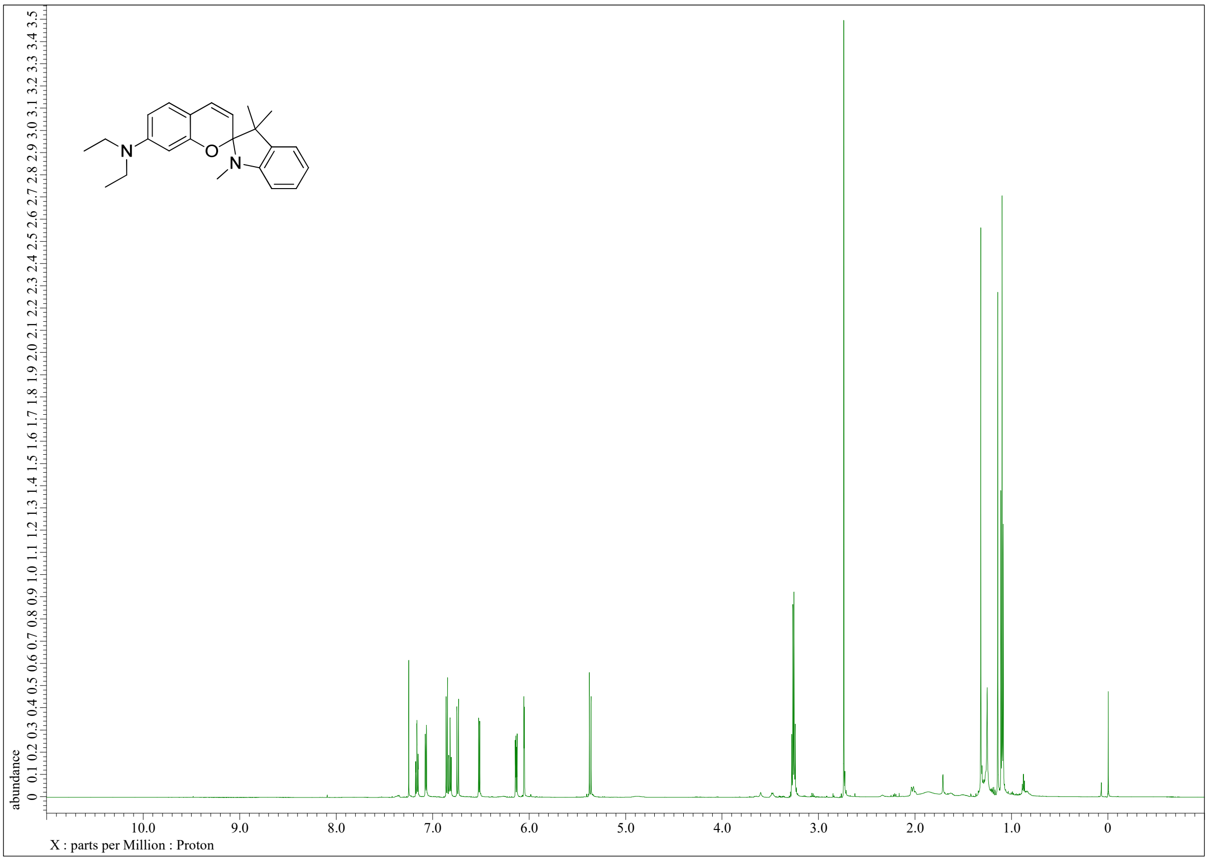

Figure S10. ^{1}H-NMR spectrum (600 MHz) of **Dye1** in $CDCl_3$.

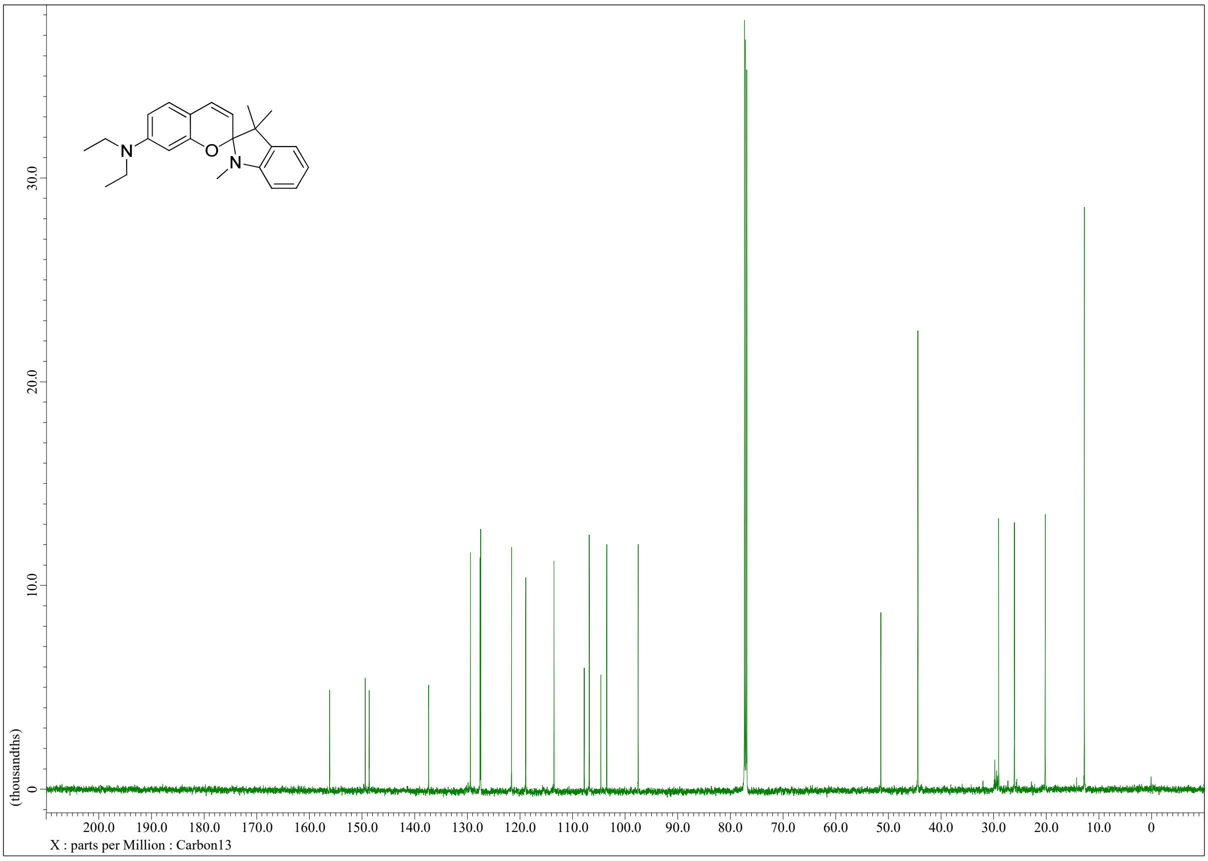

Figure S11. ^{13}C-NMR spectrum (150 MHz) of **Dye1** in $CDCl_3$.

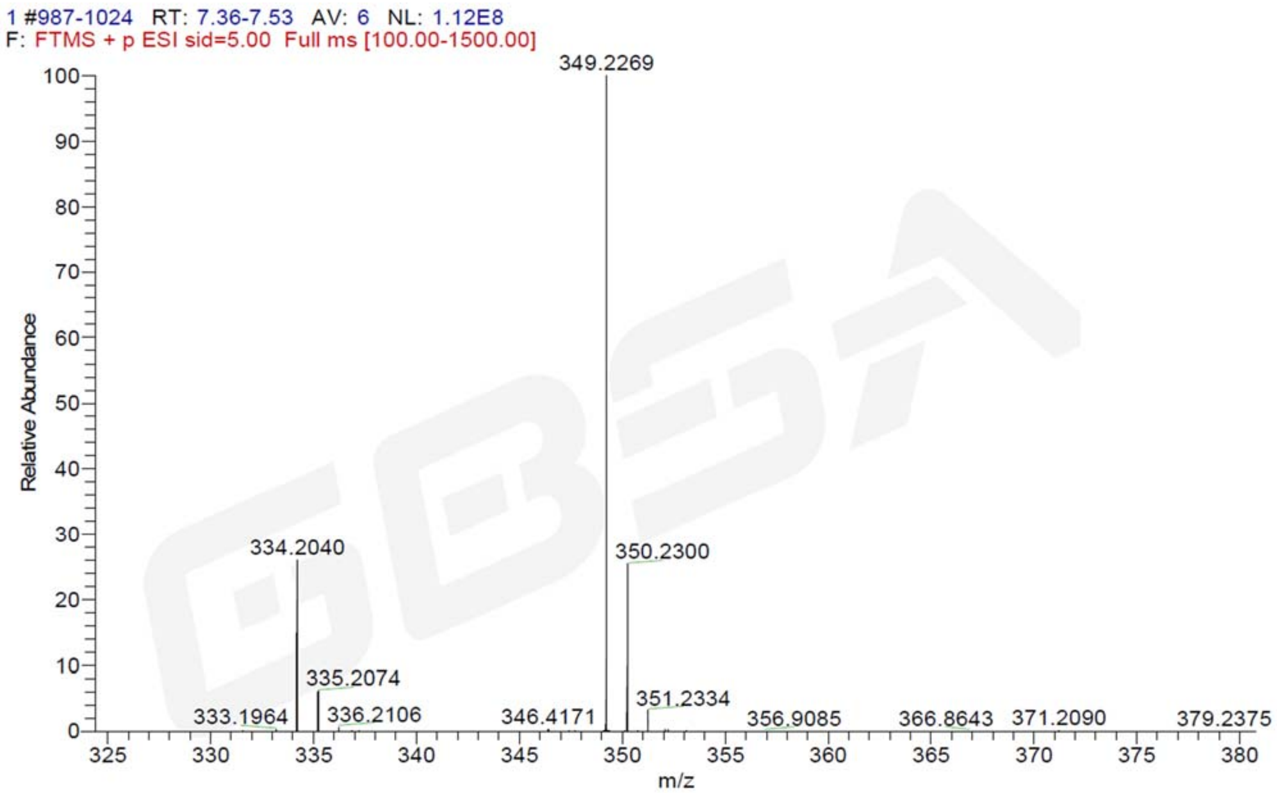

Figure S12. HRMS spectrum of **Dye1**.

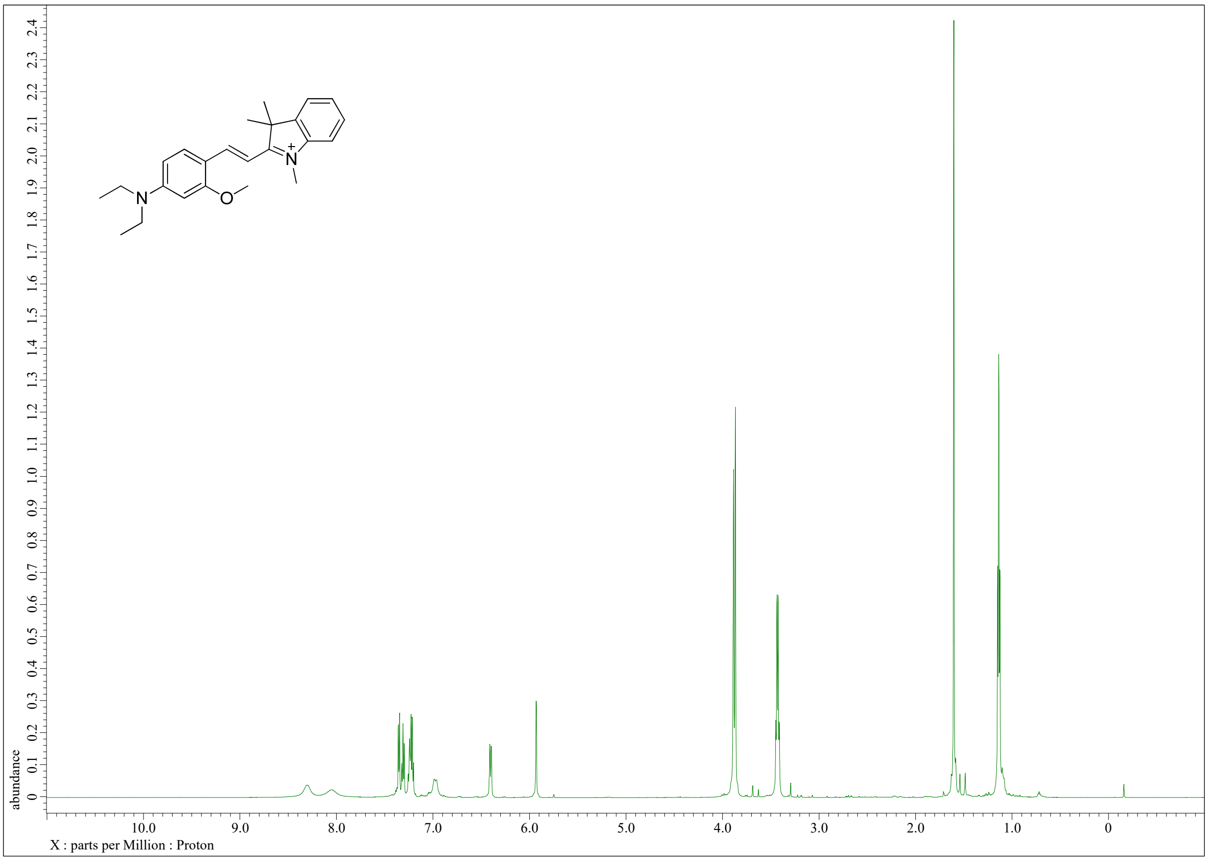

Figure S13. ^{1}H-NMR spectrum (600 MHz) of **Dye2** in $CDCl_3$.

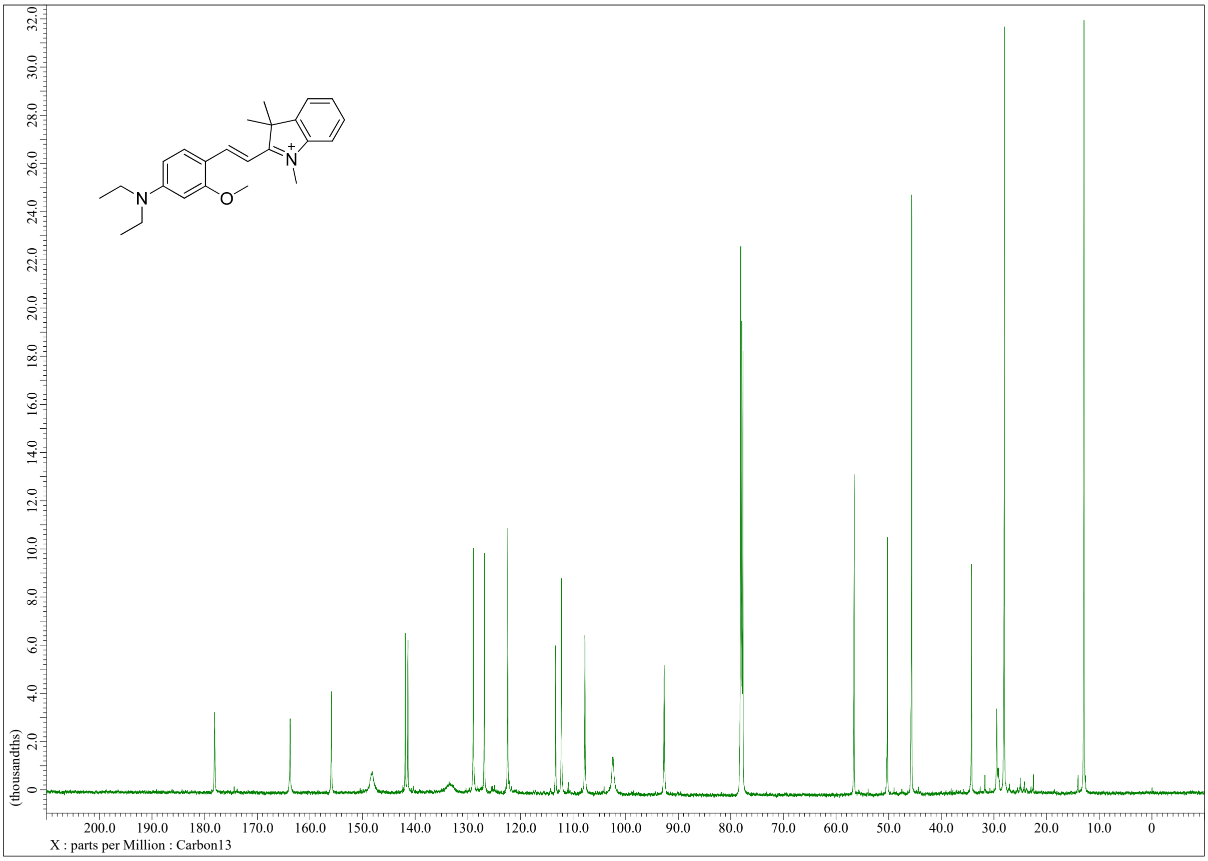

Figure S14. ^{13}C-NMR spectrum (150 MHz) of **Dye2** in $CDCl_3$.

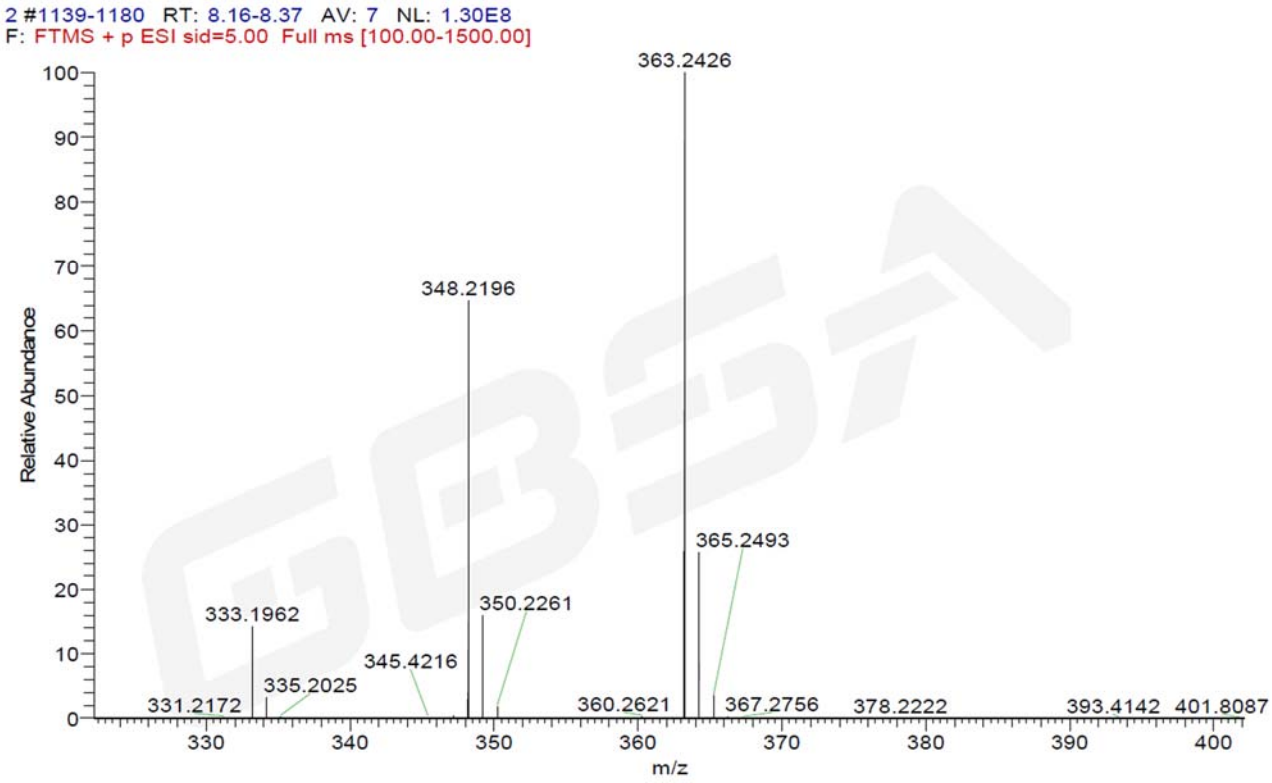

Figure S15. HRMS spectrum of **Dye2**.

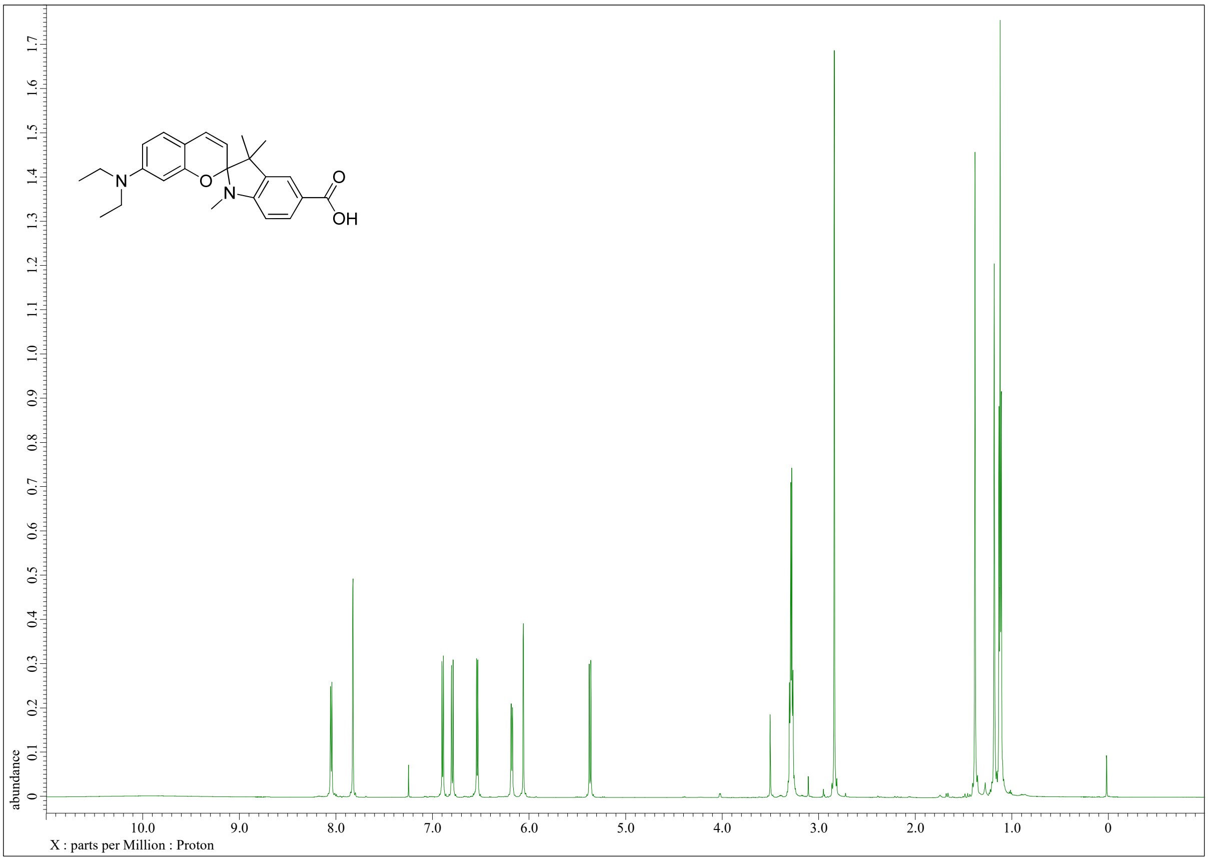

Figure S16. ^{1}H-NMR spectrum (600 MHz) of **4** in $CDCl_3$.

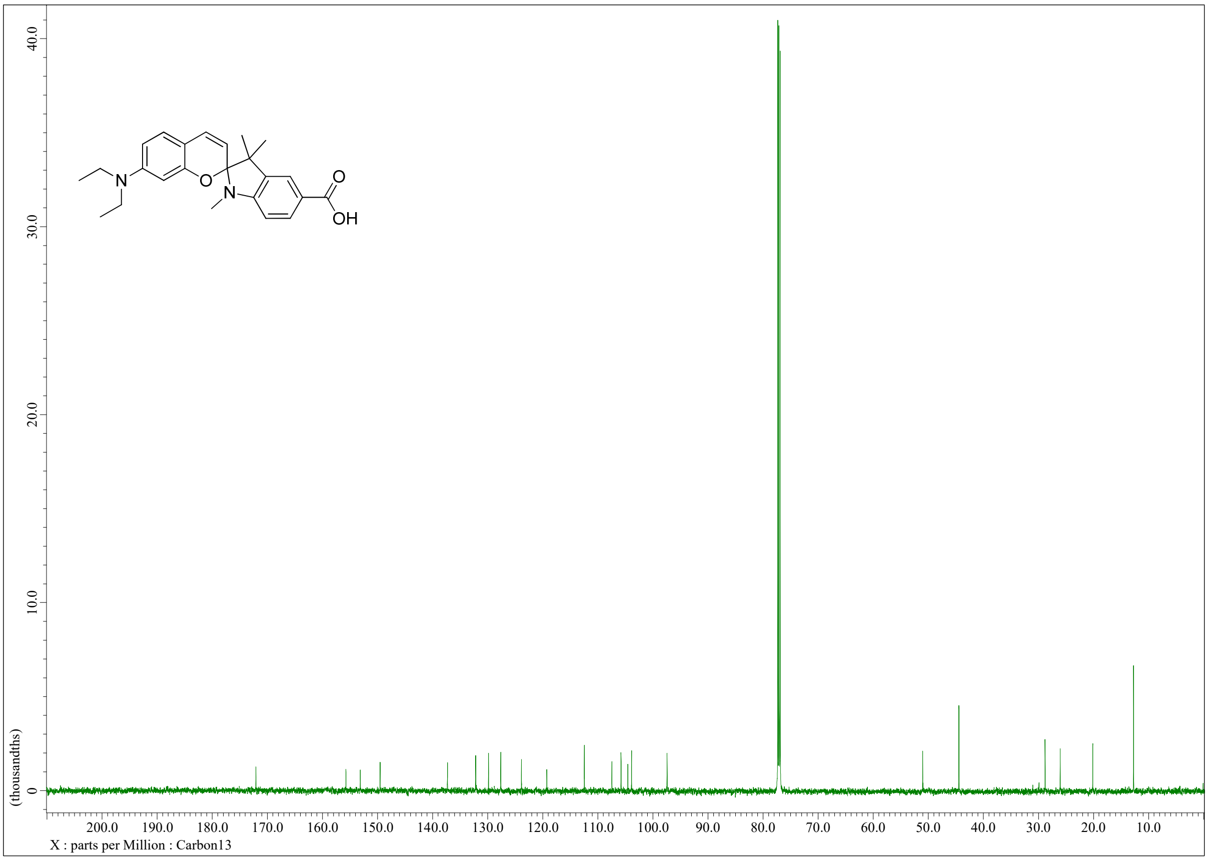

Figure S17. ^{13}C-NMR spectrum (150 MHz) of **4** in $CDCl_3$.

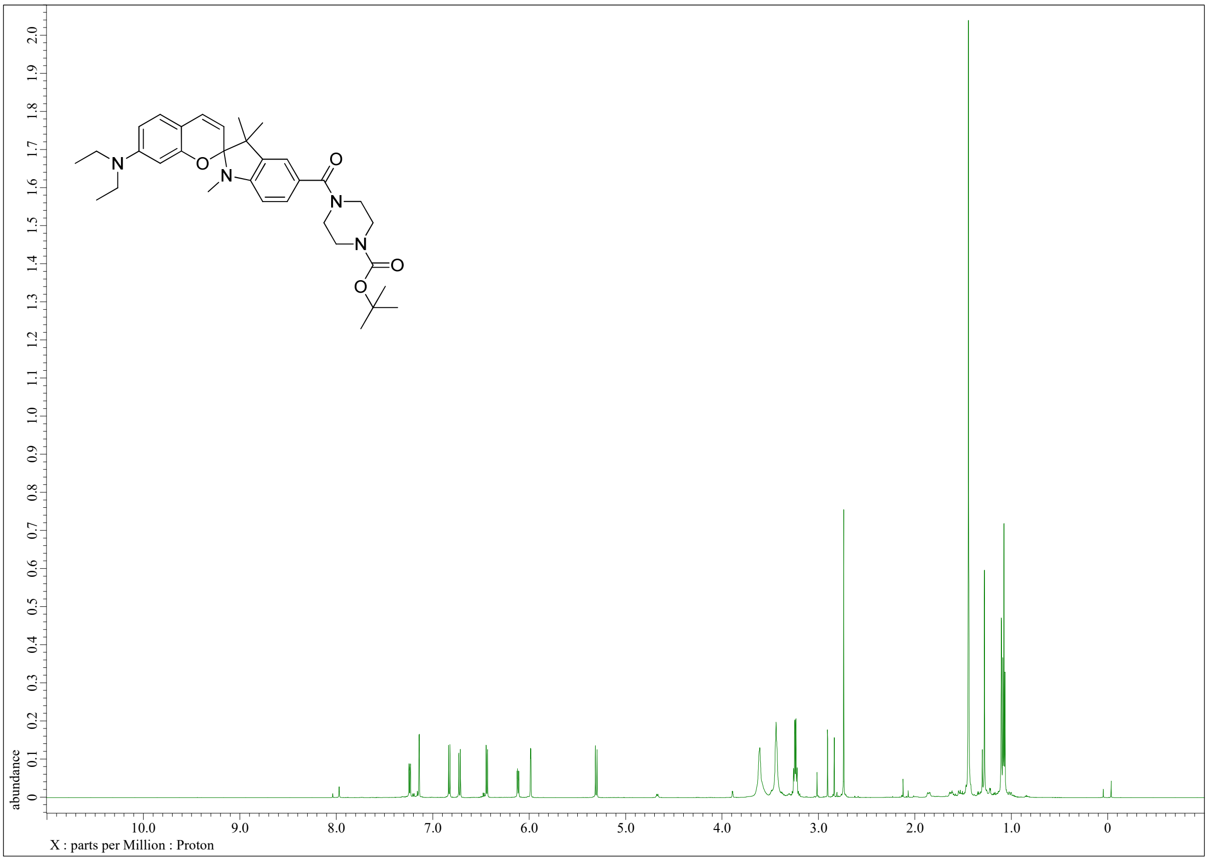

Figure S18. ^{1}H-NMR spectrum (600 MHz) of **6** in $CDCl_3$.

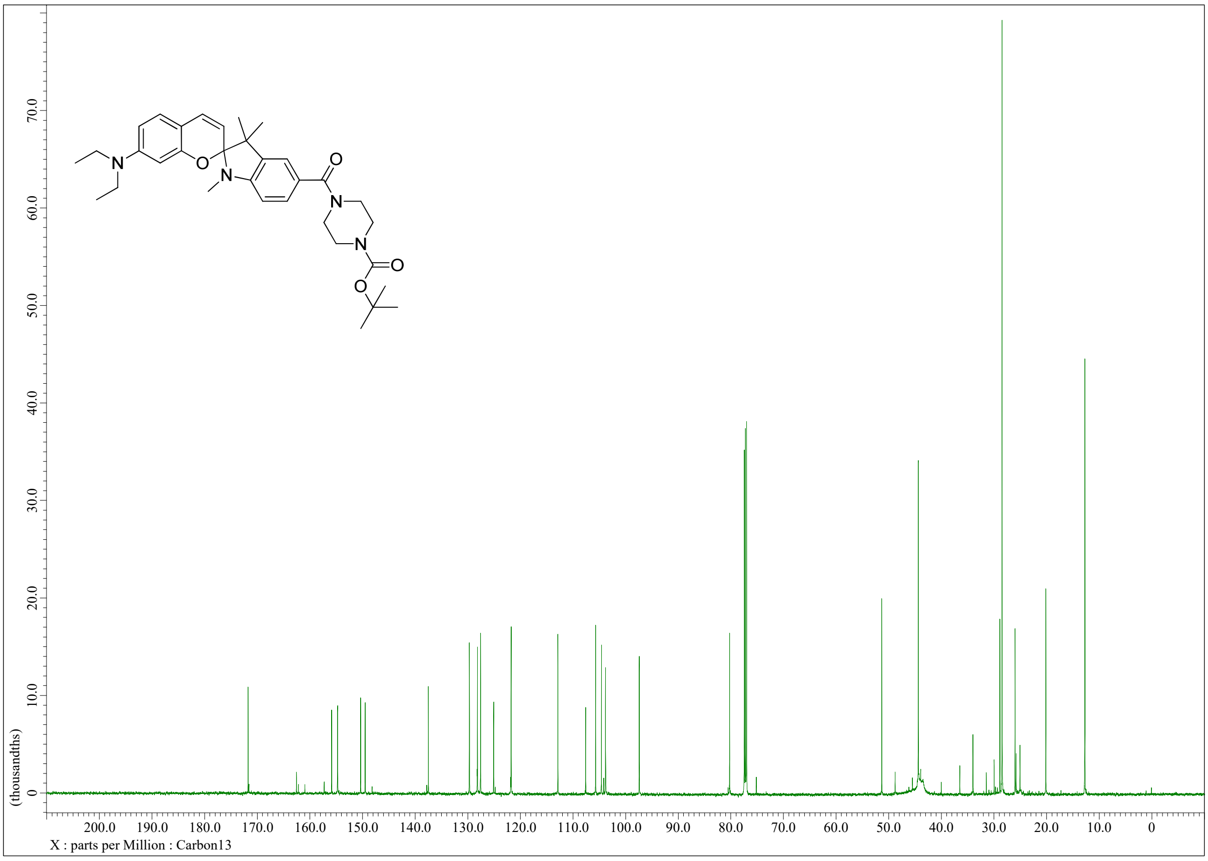

Figure S19. ^{13}C-NMR spectrum (150 MHz) of **6** in $CDCl_3$.

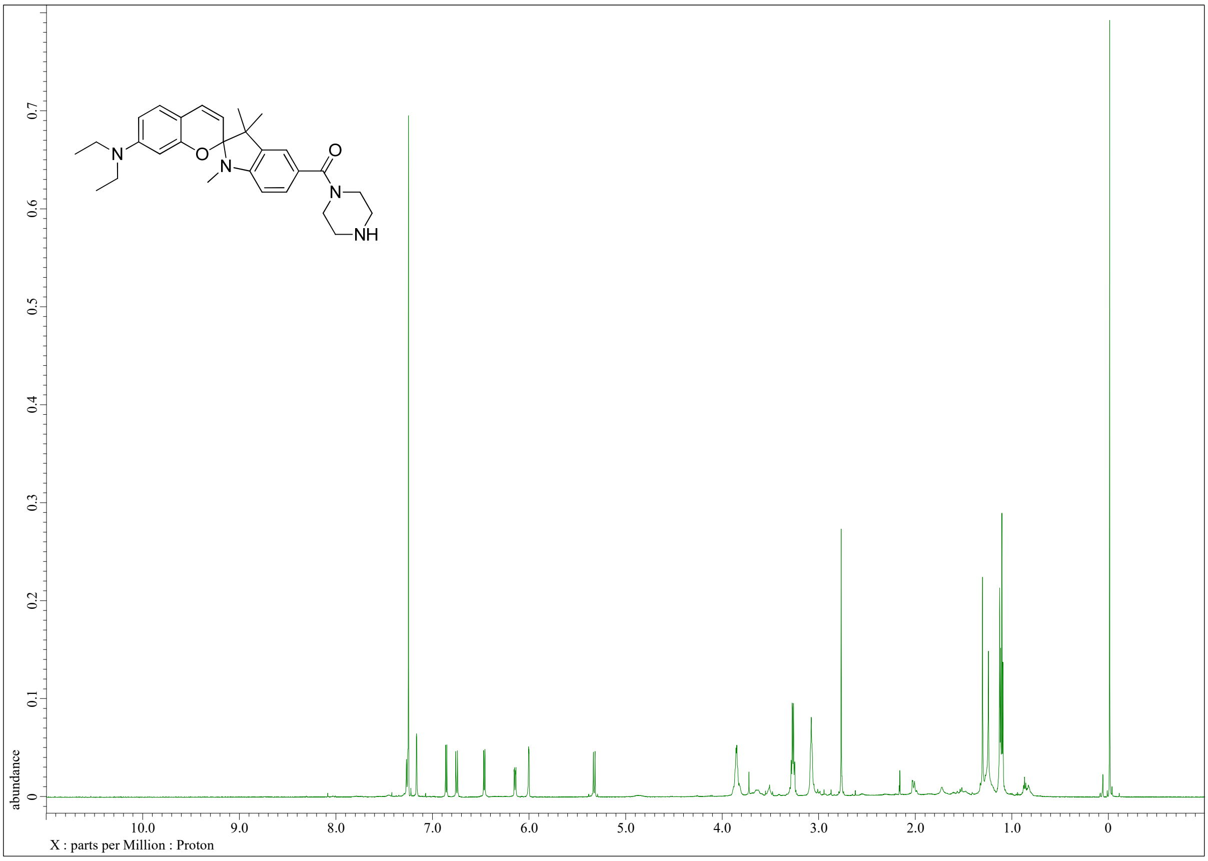

Figure S20. ^{1}H-NMR spectrum (600 MHz) of **7** in $CDCl_3$.

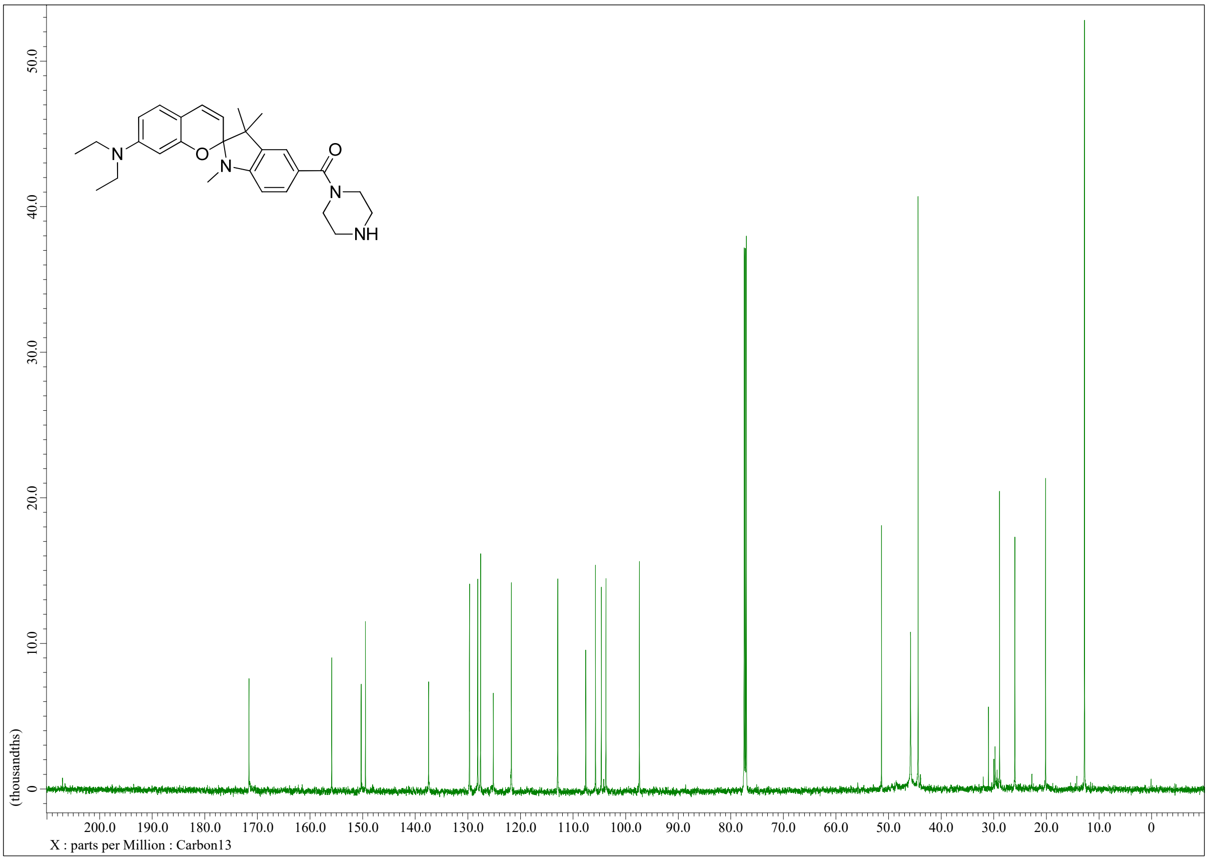

Figure S21. ^{13}C-NMR spectrum (150 MHz) of **7** in $CDCl_3$.

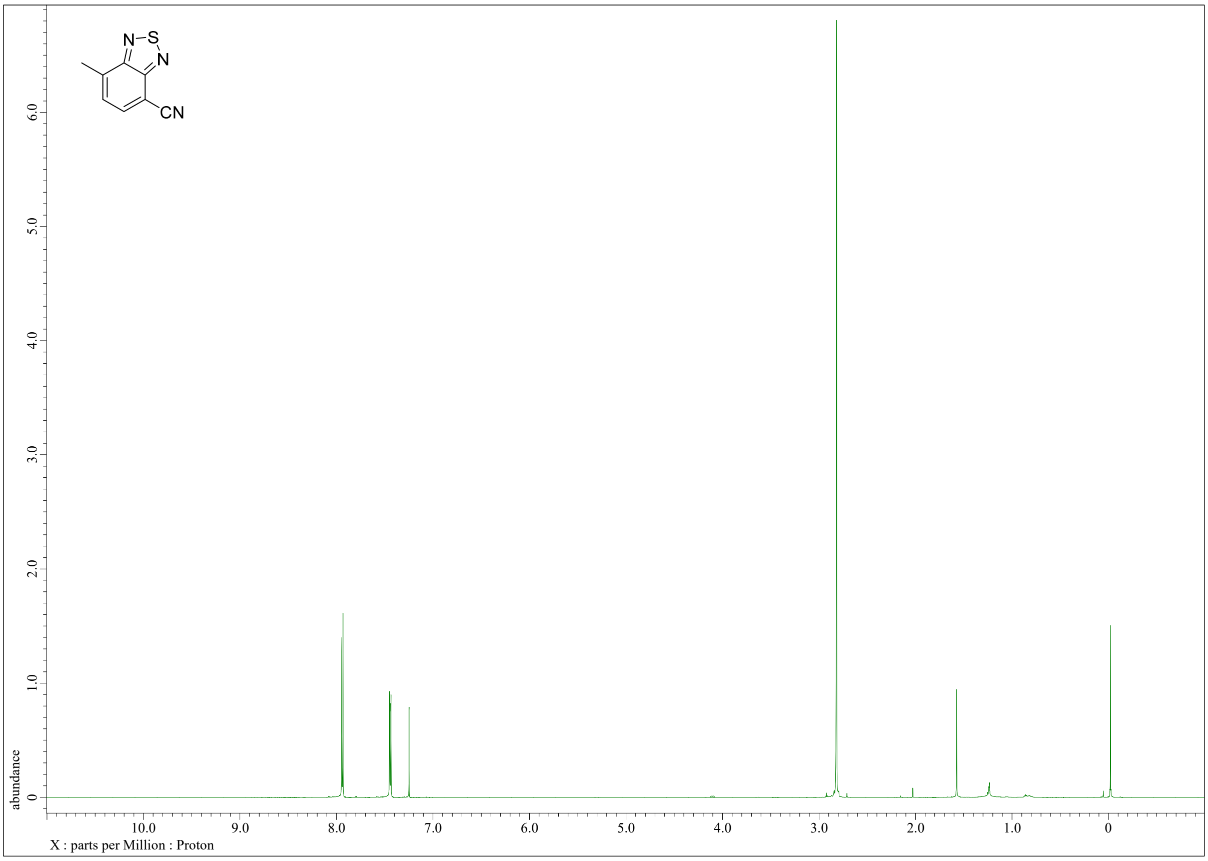

Figure S22. ^{1}H-NMR spectrum (600 MHz) of **9** in $CDCl_3$.

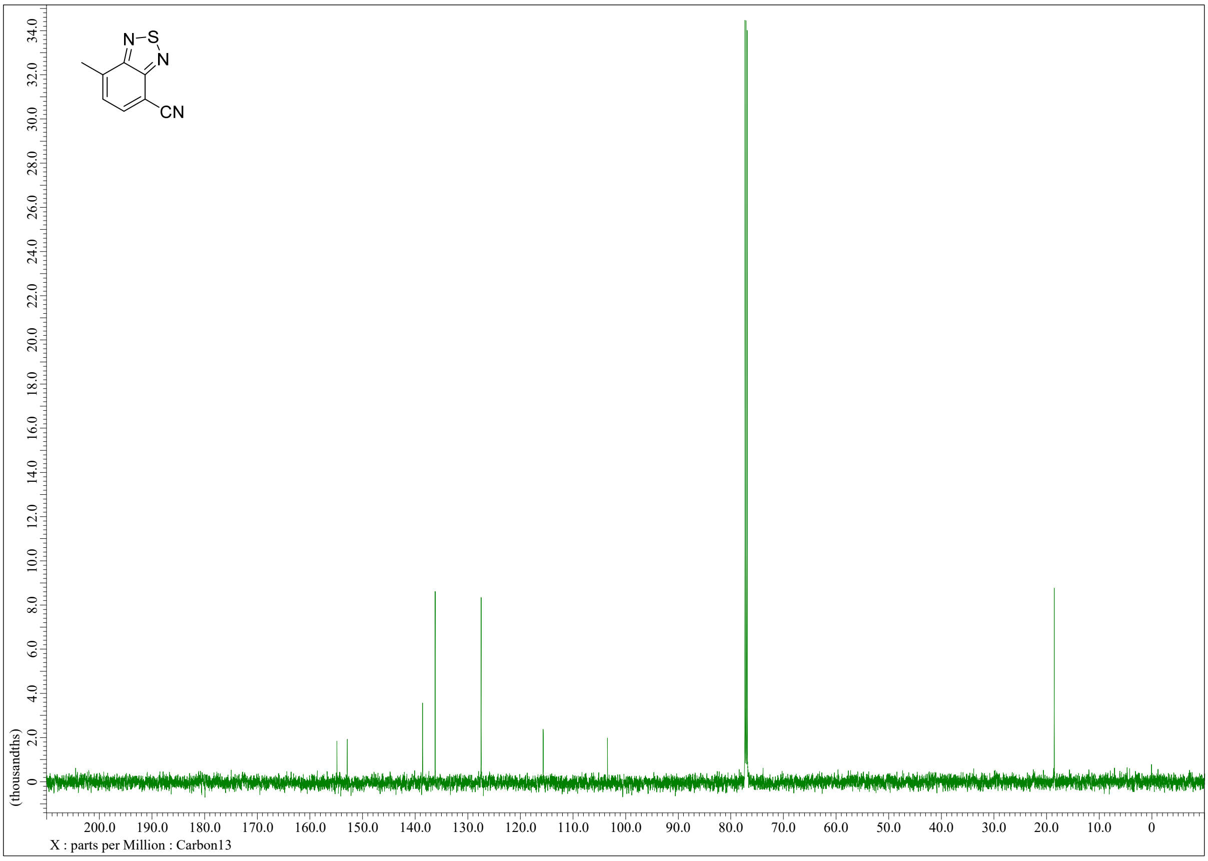

Figure S23. ^{13}C-NMR spectrum (150 MHz) of **9** in $CDCl_3$.

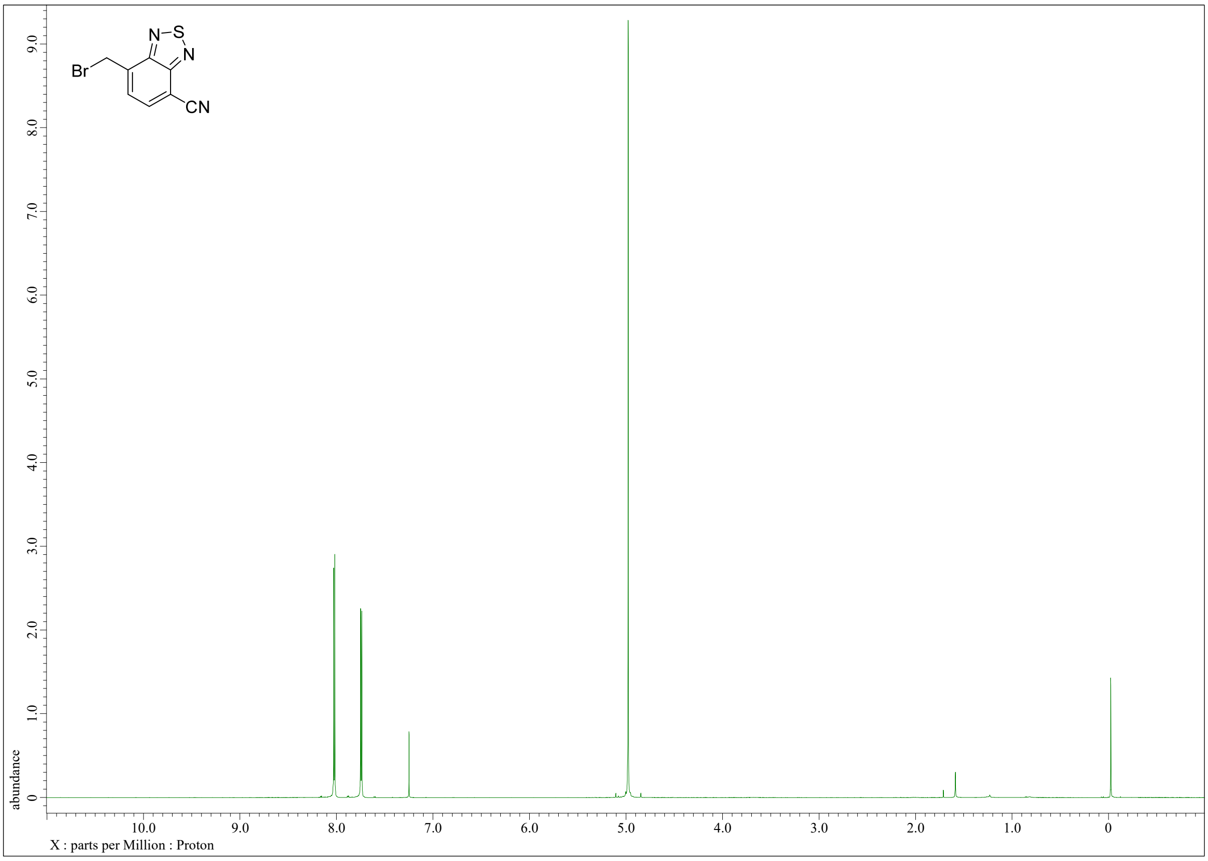

Figure S24. ^{1}H-NMR spectrum (600 MHz) of **10** in $CDCl_3$.

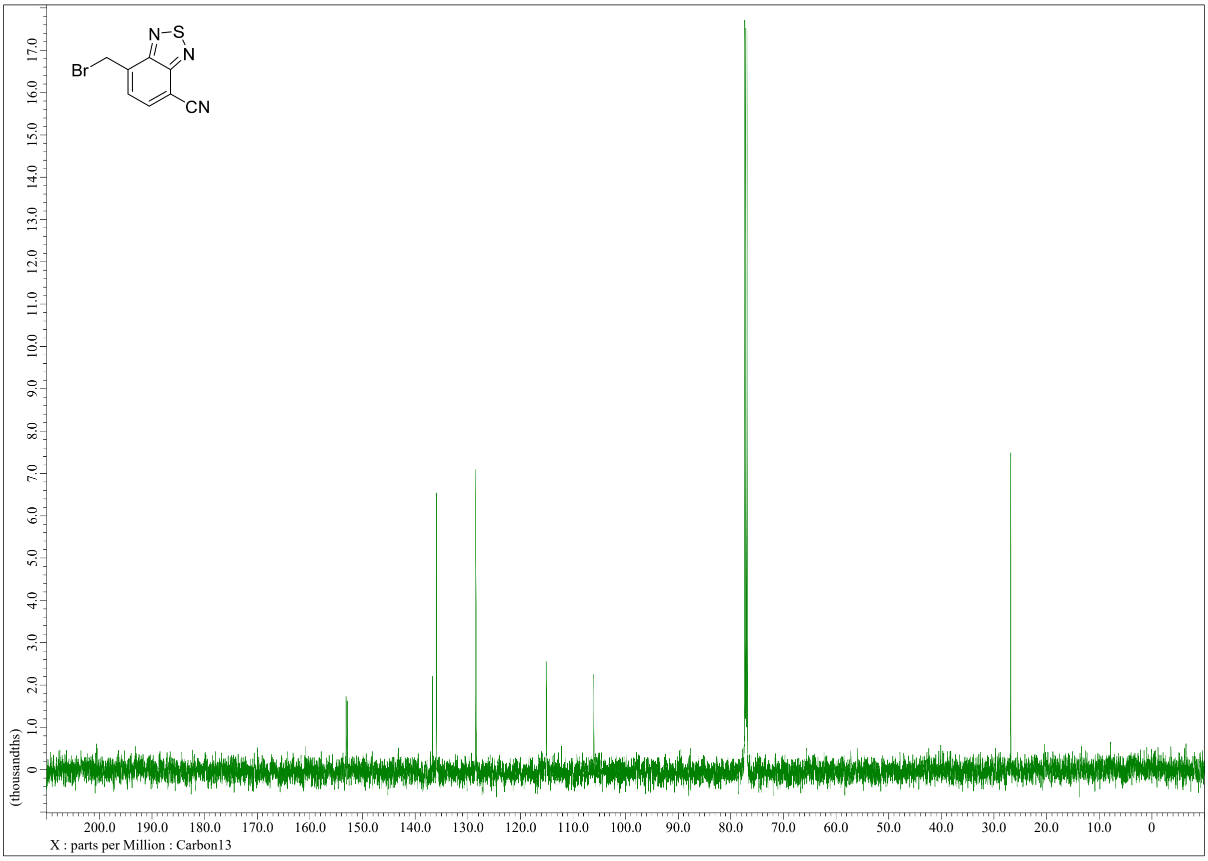

Figure S25. ^{13}C-NMR spectrum (150 MHz) of **10** in $CDCl_3$.

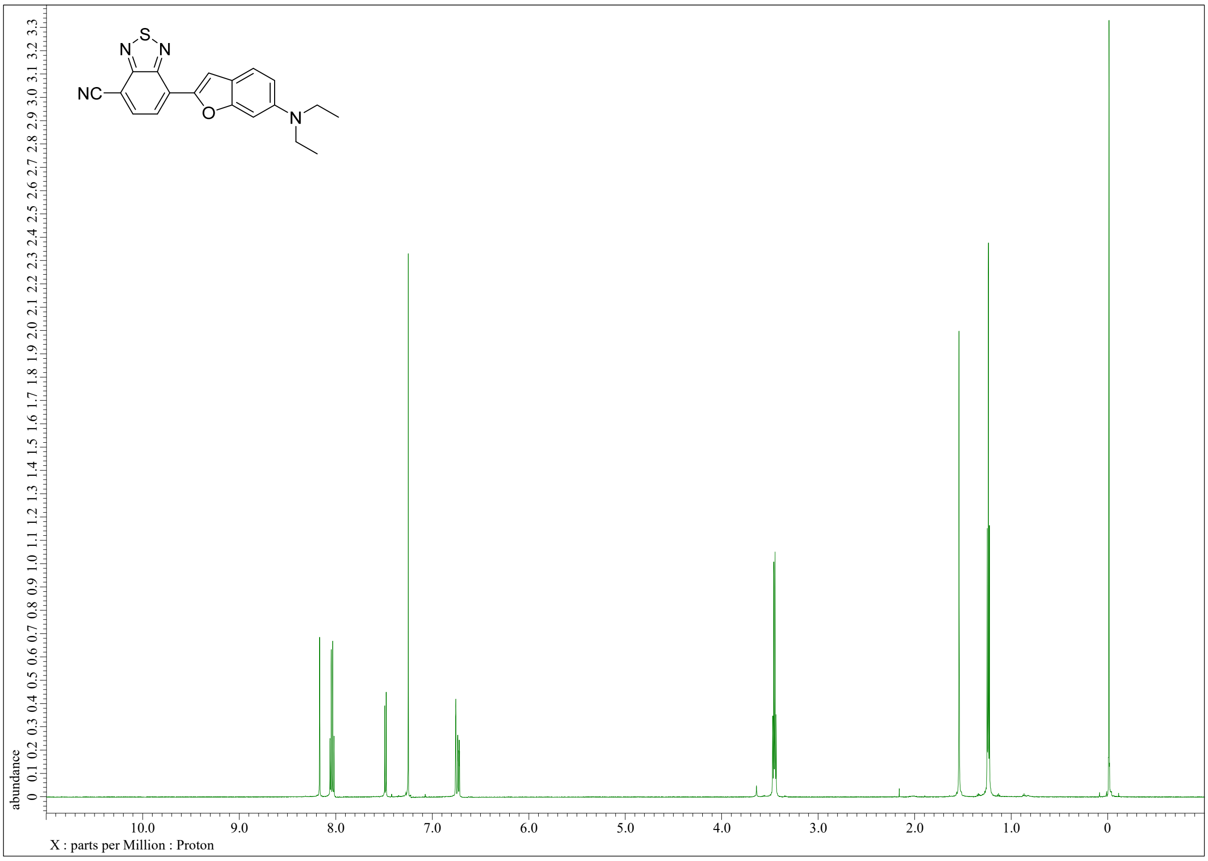

Figure S26. ^{1}H-NMR spectrum (600 MHz) of **11** in $CDCl_3$.

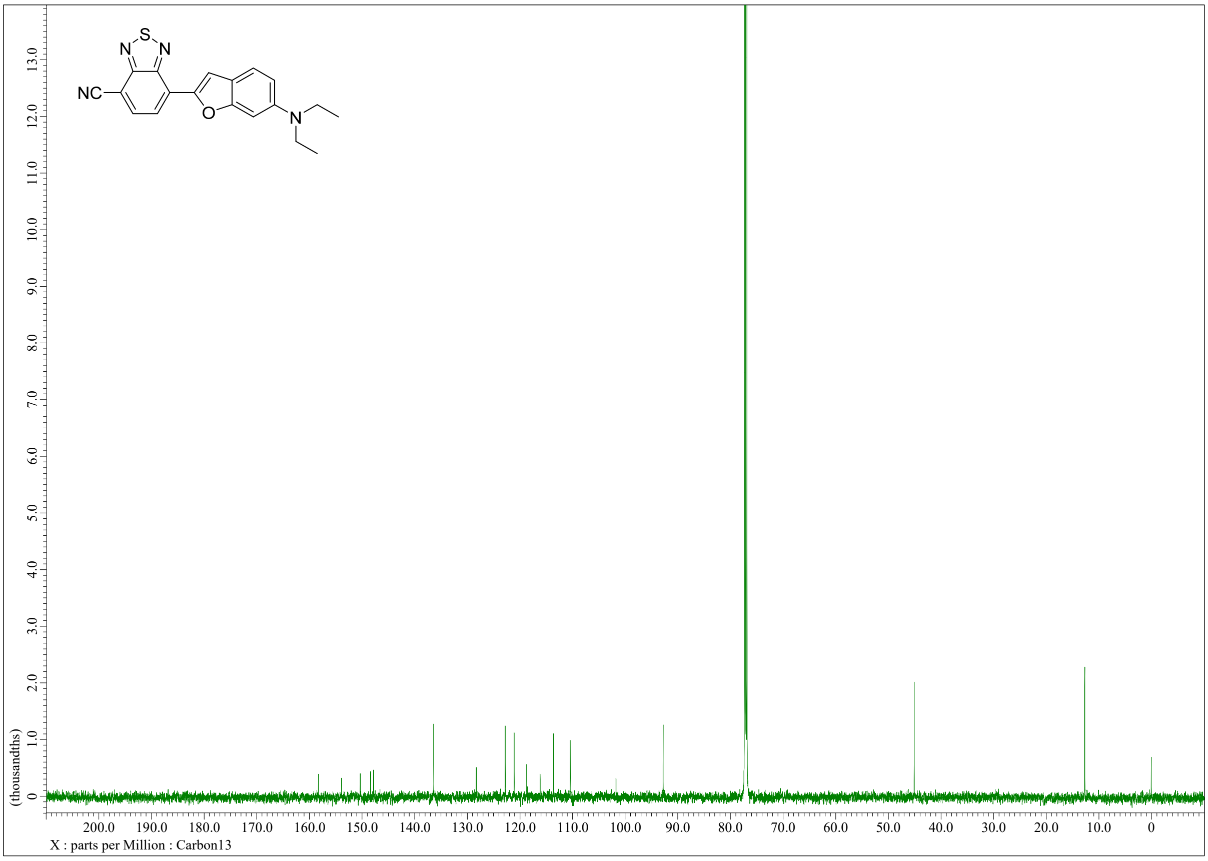

Figure S27. ^{13}C-NMR spectrum (150 MHz) of **11** in $CDCl_3$.

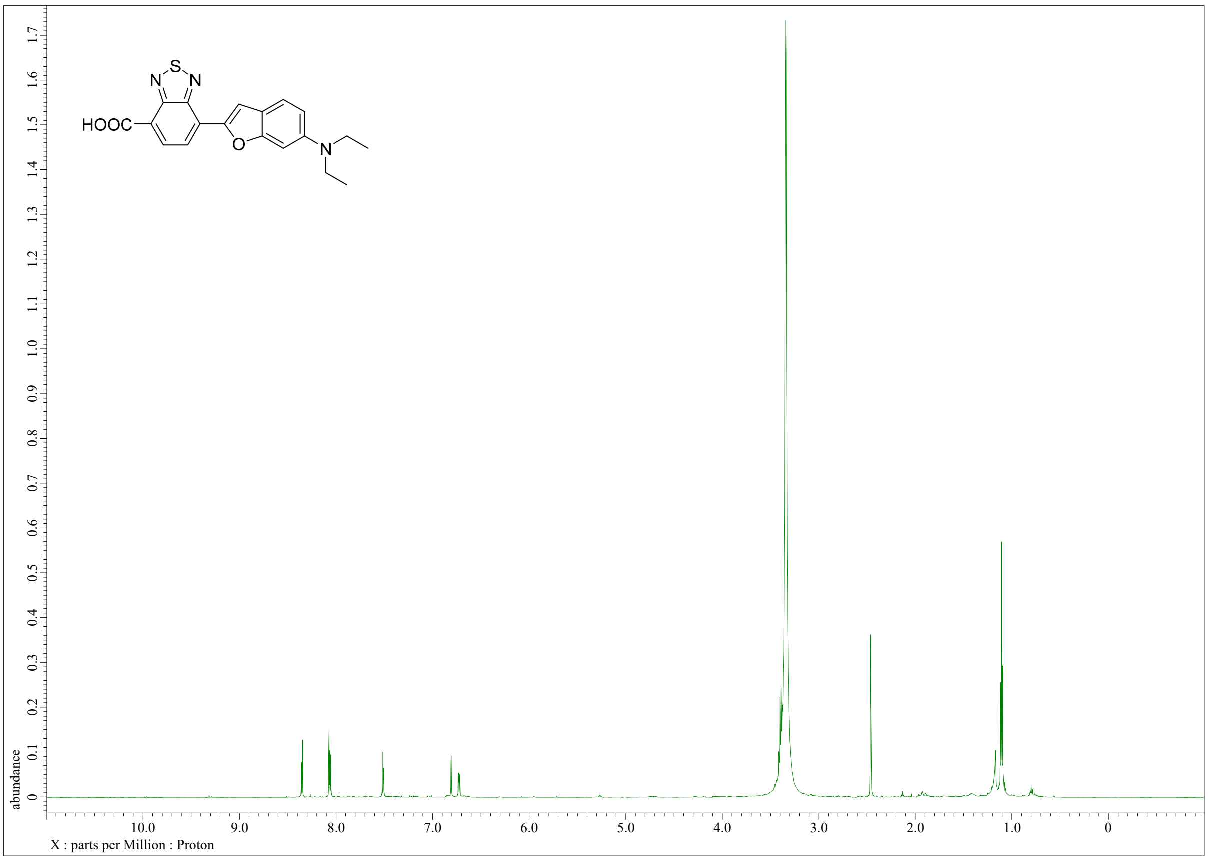

Figure S28. ^{1}H-NMR spectrum (600 MHz) of **Dye3** in DMSO.

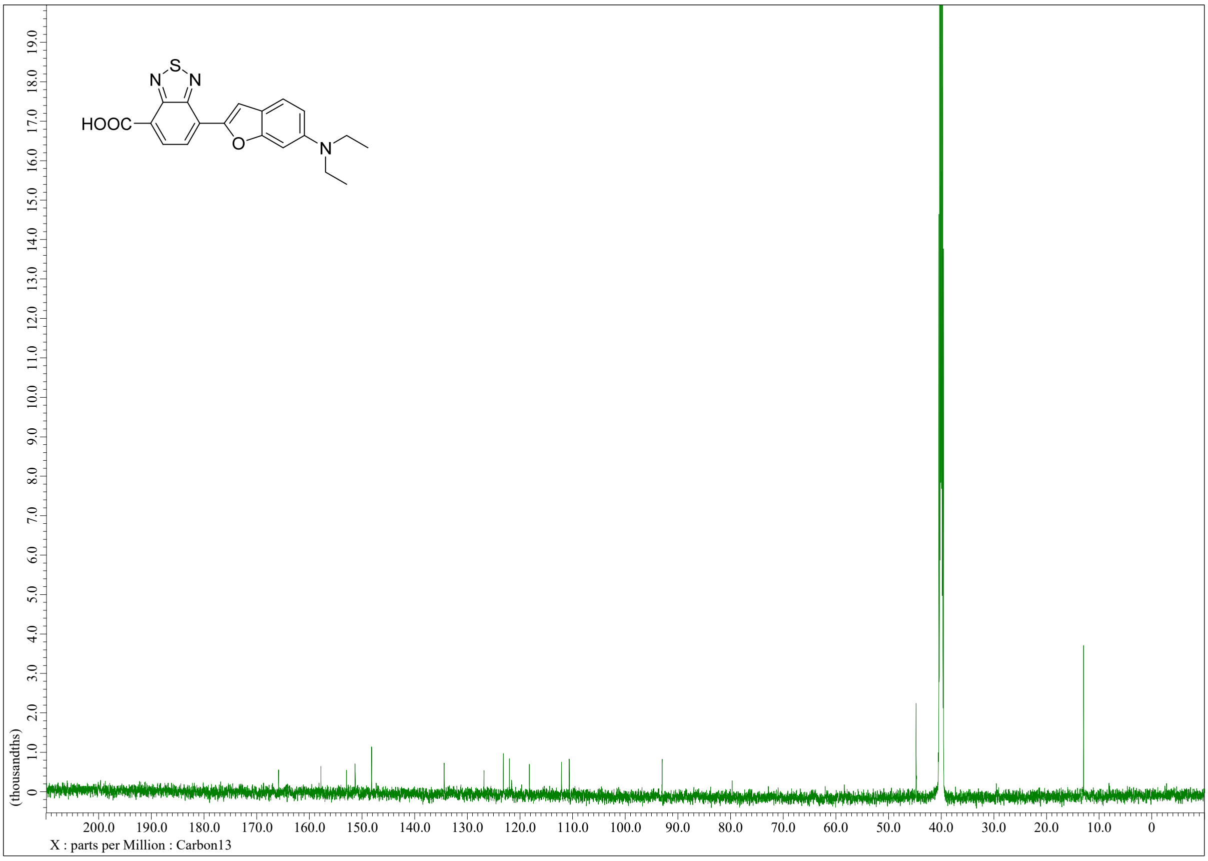

Figure S29. ^{13}C-NMR spectrum (150 MHz) of **Dye3** in DMSO.

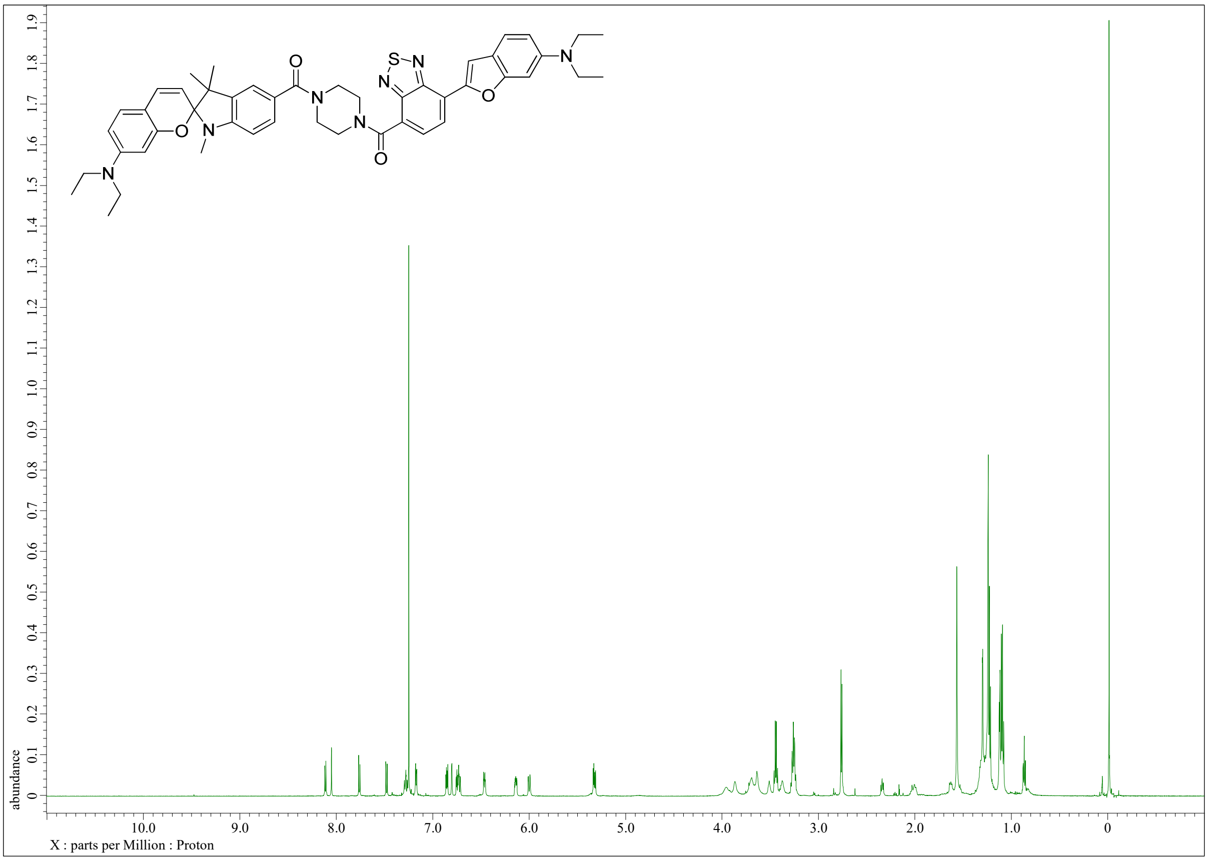

Figure S30. ^{1}H-NMR spectrum (600 MHz) of **RPS-1** in $CDCl_3$.

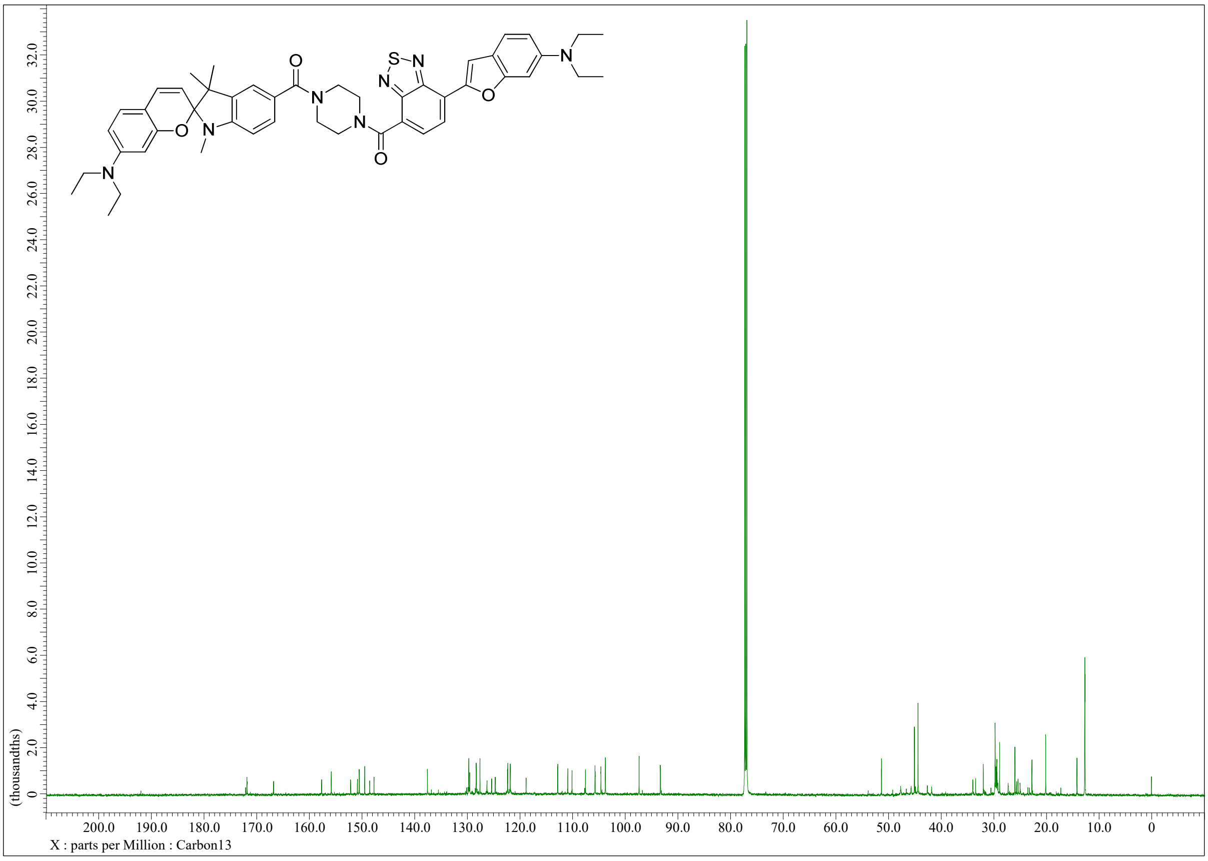

Figure S31. ^{13}C-NMR spectrum (150 MHz) of **RPS-1** in $CDCl_3$.

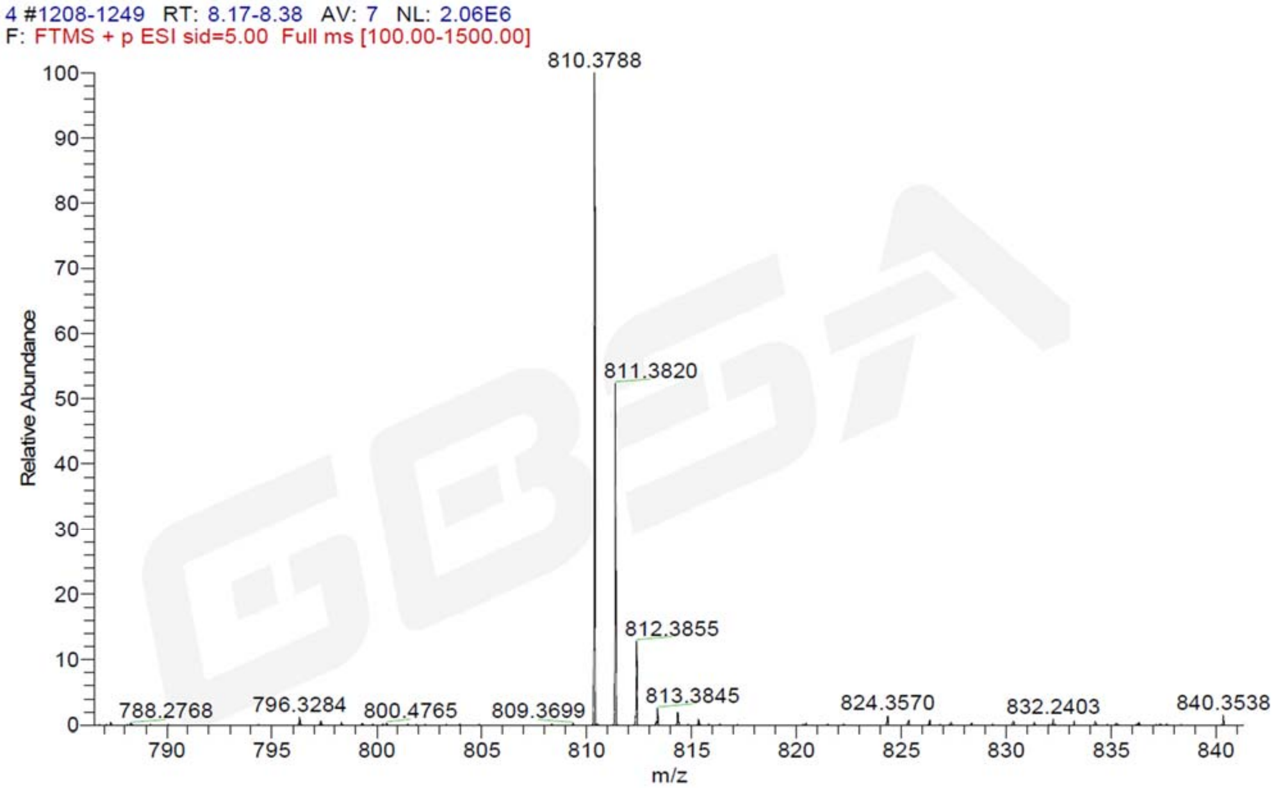

Figure S32. HRMS spectrum of **RPS-1**.

References

(1) X. Song, H. Bian, C. Wang, M. Hu, N. Li and Y. Xiao, *Org. biomol. Chem.,* 2017, **15**, 8091–8101.

(2) P. Chou, C. Huang, S. Pu, Y. Cheng, Y. Liu, Y. Wang, and C. Chen, *J. Phys. Chem. A,* 2004, **108**, 6452–6454.

(3) A. Nano, A. N. Boynton and J. K. Barton, *J. Am. Chem. Soc.,* 2017, **139**, 17301–17304.

(4) B. Biannic and J. J. Bozell, *Org. Lett.,* 2013, **15**, 2730–2733.

(5) R. Agneeswari, V. Tamilavan, M. Song and M. H. Hyun, *J. Mater. Chem. C,* 2014, **2**, 8515–8524.

(6) M. Uesugi, T. Hayashi and H. E. Jasin, *J. Immunol.* 1998, **161**, 1422–1427.
